# The circSPON2/miR-331-3p axis regulates PRMT5, an epigenetic regulator of CAMK2N1 transcription and prostate cancer progression

**DOI:** 10.1186/s12943-022-01598-6

**Published:** 2022-05-27

**Authors:** Bing Yao, Sha Zhu, Xiyi Wei, Ming-Kun Chen, Yangkun Feng, Zhimin Li, Xinyu Xu, Yuwei Zhang, Yang Wang, Jingwan Zhou, Ningyuan Tang, Chengjian Ji, Peng Jiang, Shan-Chao Zhao, Chao Qin, Ninghan Feng

**Affiliations:** 1 Department of Urology, The Affiliated Wuxi No. 2 People’s Hospital of Nanjing Medical University, Department of Medical Genetics, Nanjing Medical University, Nanjing, 211166 China; 2 The State Key Lab of Reproductive Medicine, Department of Urology, The First Affiliated Hospital of Nanjing Medical University, Nanjing, 210029 China; 3 Department of Urology, the Third Affiliated Hospital of Southern Medical University, Guangzhou, 510500 China; 4 Medical School of Nantong University, Nantong, 226001 China; 5 Department of Urology, Nanfang Hospital, Southern Medical University, Guangzhou, 510515 China; 6 Wuxi Clinical College, Nantong University, Wuxi, 214002 China

**Keywords:** PRMT5, CAMK2N1, circSPON2, miR-331-3p, Prostate cancer

## Abstract

**Background:**

Prostate cancer (PCa) is the most frequently diagnosed malignancy in men, and its mechanism remains poorly understood. Therefore, it is urgent to discover potential novel diagnostic biomarkers and therapeutic targets that can potentially facilitate the development of efficient anticancer strategies.

**Methods:**

A series of functional *in vitro* and *in vivo* experiments were conducted to evaluate the biological behaviors of PCa cells. RNA pulldown, Western blot, luciferase reporter, immunohistochemistry and chromatin immunoprecipitation assays were applied to dissect the detailed underlying mechanisms. High-throughput sequencing was performed to screen for differentially expressed circRNAs in PCa and adjacent normal tissues.

**Results:**

Upregulation of protein arginine methyltransferase 5 (PRMT5) is associated with poor progression-free survival and the activation of multiple signaling pathways in PCa. PRMT5 inhibits the transcription of CAMK2N1 by depositing the repressive histone marks H4R3me2s and H3R8me2s on the proximal promoter region of CAMK2N1, and results in malignant progression of PCa both *in vitro* and *in vivo*. Moreover, the expression of circSPON2, a candidate circRNA in PCa tissues identified by RNA-seq, was found to be associated with poor clinical outcomes in PCa patients. Further results showed that circSPON2 induced PCa cell proliferation and migration, and that the circSPON2-induced effects were counteracted by miR-331-3p. Particularly, circSPON2 acted as a competitive endogenous RNA (ceRNA) of miR-331-3p to attenuate the repressive effects of miR-331-3p on its downstream target PRMT5.

**Conclusions:**

Our findings showed that the epigenetic regulator PRMT5 aggravates PCa progression by inhibiting the transcription of CAMK2N1 and is modulated by the circSPON2/miR-331-3p axis, which may serve as a potential therapeutic target for patients with aggressive PCa.

**Supplementary Information:**

The online version contains supplementary material available at 10.1186/s12943-022-01598-6.

## Introduction

Prostate cancer (PCa) is one of the most diagnosed visceral malignancy and the second leading cause of cancer-associated death in men [1]. According to GLOBOCAN statistics, there were 1.3 million of new estimated cases of PCa and approximately 359,000 PCa-associated deaths in 2018 globally [2]. Lack of knowledge on the mechanisms of PCa tumorigenesis and the absence of potential target therapies for PCa, especially high-grade PCa, may contribute to the poor survival rate of patients with PCa [3]. Therefore, understanding the pathophysiological mechanisms involved in PCa progression is needed to discover potential novel diagnostic biomarkers that can facilitate the development of highly efficient therapies.

Arginine methylation governs a variety of cellular events that modulate growth and proliferation, differentiation, senescence, apoptosis and tumorigenesis [4]. Initially identified as a JAK-binding protein 1, protein arginine methyltransferase 5 (PRMT5) transfers methyl groups from S-adenosylmethionine (SAM) to a guanidine nitrogen of protein arginine, generating a methylated guanidinium moiety and S-adenosylhomocysteine (SAH) [5]. PRMT5 is a type II protein arginine methyltransferase that catalyzes symmetric di-methylation (me2s) of on H2AR3, H3R2, H3R8, and H4R3, which is generally associated with transcriptional repression [6, 7]. As an important epigenetic regulator, PRMT5 associates with chromatin remodelers, co-repressors, and co-activators, and regulates the transcription of tumor suppressor genes, such as ST7, RBL2, CCNE, and CDH1 [4]. How PRMT5 impacts tumorigenesis and cancer progression through its regulation of gene expression is not well defined.

Circular RNAs (circRNAs) are an emerging class of endogenous, abundant, single-stranded non-coding RNA molecules characterized by a covalently closed loop structure (without 5’-methylguanylate cap or 3’-polyadenylate tail) derived from exons by an alternative mRNA splicing known as back-splicing [8, 9]. CircRNAs were initially thought to be spurious transcriptional “noise”, without significant biological functions in eukaryotic cells [10]. Following the rapid development of high-throughput sequencing (HTS) technologies and bioinformatics analyses, an increasing number of circRNAs have been discovered across the eukaryotic lineage and have become research hotspots in the field of RNA biology [11, 12]. Recent studies have suggested that dysregulated circRNAs are involved in the tumorigenesis, progression, and metastasis of a variety of human malignancies [13, 14], such as lung [15], gastric [16], colorectal [17], breast [18], glioma [19], and other types of carcinomas. CircRNAs have diverse mechanisms in regulation of various cancers: acting as microRNA (miRNA) sponges, regulating transcription, functioning as competing elements that block protein activities, and translating proteins in a cap-independent manner [20, 21]. However, our knowledge of circRNAs remains preliminary.

Here, we show that epigenetic regulator PRMT5, which is modulated by the circSPON2/miR-331-3p axis, promotes the progression of PCa through inhibiting the transcription of CAMK2N1. The novel circSPON2/miR-331-3p/PRMT5/CAMK2N1 axis may serve as a promising therapeutic target of PCa.

## Materials and methods

### Human subjects

A total of 248 PCa tissues and adjacent normal tissues were recruited into the study cohort from the First Affiliated Hospital of Nanjing Medical University (Supplementary Table S1). Written informed consent was obtained from all patients and the study was approved by the Ethics Committee of the First Affiliated Hospital of Nanjing Medical University.

### Cell lines and cell culture

The human normal embryonic kidney cell line HEK-293 T, human normal prostate epithelial cell line RWPE-1, and PCa cell lines (PC-3, DU145, LNCaP, VCaP and 22RV1) were purchased from the Cell Bank of the Chinese Academy of Sciences (Shanghai, China). The HEK 293 T and VCaP cells were cultured in Dulbecco’s Modified Eagle’s/Eagle Medium (DMEM; Gibco). The RWPE-1 cells were cultured in Keratinocyte Serum-free Medium (K-SFM; Gibco). PC-3, DU145, LNCaP, and 22RV1 cells were cultured in Roswell Park Memorial Institute medium (RPMI-1640; Gibco). All these cell lines were cultured in medium containing 10% fetal bovine serum (FBS; Gibco), 100 U/mL penicillin and 100 μg/mL streptomycin (Invitrogen) at 37 °C in a humidified incubator gassed with 95% air and 5% CO_2_.

### Phospho-specific protein microarray

We used a phospho-specific antibody microarray (CSP100_Plus, Full Moon BioSystems, USA) containing 304 antibodies against 157 phosphorylated sites and 144 unphosphorylated sites. The antibody array experiment was performed by Wayen Biotechnology (Shanghai, China) according to the manufacturer’s instructions. The SureScan Dx Microarray Scanner was used to scan for chip images, and the raw data were acquired using GenePix Pro 6.0 software. The phosphorylation ratio of each protein was calculated according to the formula: phosphorylation ratio = phospho value/unphospho value.

### Plasmid construction

The circSPON2 overexpression plasmid (oe-circSPON2) was generated by inserting the full-length circSPON2 sequence into the pLCDH-ciR lentiviral vector. The miR-331-3p overexpression plasmid (oe-miR-331-3p) was fabricated by inserting the pre-miR-331-3p sequence into the pLL3.7 lentiviral vector. The PRMT5 overexpression plasmid (oe-PRMT5) was constructed by inserting the full-length of PRMT5 sequence into the pLVX lentiviral or pcDNA3.1 vectors. The shRNA sequences were designed by Designer of Small Interfering RNA and chemically synthesized by Shanghai GenePharma (Shanghai, China). shRNA lentiviral plasmids targeting circSPON2 (circSPON2 shRNAs), PRMT5 (sh_PRMT5), or CAMK2N1 (sh_CAMK2N1) were constructed by inserting annealed shRNA template DNA sequences into the pLKO.1-TRC-puromycin vector. To construct luciferase reporter plasmids, the sequence of wild-type (WT) circSPON2, miR-331-3p binding site mutated circSPON2 (Mut), wild-type (WT) PRMT5 3’-UTR, and miR-331-3p binding site mutated PRMT5 3’-UTR (Mut) were cloned into the psiCHECK-2 vector, respectively. The promoter sequence of CAMK2N1 (-2000 to -1 bp region upstream of the TSS) was cloned into the pGL3.0-Basic vector. The sequences of shRNAs are provided in Supplementary Table S2.

### Cell transfection

For transfection, cells were seeded in 6-well plates and grown to 40–60% confluence by the time of transfection. MiR-331-3p, miR-331-3p inhibitor or NC mimics, and constructed plasmids or empty vectors were transfected with Lipofectamine™ 3000 reagent (Invitrogen, USA) according to the manufacturer’s instructions.

### Lentivirus package and infection

For the lentivirus package, the core lentivirus expression plasmid pLCDH-ciR, pLL3.7, pLKO.1-TRC-shRNA, or pLVX, and the psAX2 packaging plasmid and pMD2.G envelope plasmid were transiently co-transfected into HEK-293 T cells using Lipofectamine™ 3000 reagent (Invitrogen, USA) to produce the lentivirus. After 48 h post transfection, the cultured lentivirus supernatants were collected and filtered through cellulose acetate filters (Millipore; 0.22 μm pore size). The PCa cell lines were then infected by the recombinant lentivirus carrying shRNAs or the negative control shRNA (NC) in the presence of hexadimethrine bromide (Polybrene; Beyotime; C0351) for 48 h, and puromycin dihydrochloride (Sigma; 540,222) was used to select the stable transfected cell lines.

### Cell Counting Kit-8 (CCK-8) assay

Cell proliferation was measured by using CCK-8 Cell Counting Kit (Vazyme; A311-01) according to the manufacturer’s instructions. Briefly, cells were seeded onto plastic 96-well plates at an initial density of 2 × 10^3^ cells/well. Then, CCK8 solution was added to each well at the indicated times and incubated for an additional 2 h at 37 °C. Thereafter, OD450 values were measured using a microplate reader (Model 550, Bio‐Rad, Laboratories, Inc).

### 5-Ethynyl-20-deoxyuridine (EdU) incorporation assay

The EdU (5-ethynyl-2’-deoxyuridine) incorporation assay was performed to detect DNA synthesis (an indicator of cell proliferation) by using a Cell-Light EdU Apollo 488 In Vitro kit (RiboBio, C10310-3) following the manufacturer’s protocol. Briefly, cells were incubated with EdU (50 nM), fixed with 4% paraformaldehyde fix solution (Beyotime; P0099), permeabilized with 0.5% Triton X-100, and stained with 1 × Apollo® fluorescent dyes. Cell nuclei were stained blue using 1 × Hoechst 33,342 (1:1000). The EdU positive cells were photographed and counted under an Olympus FSX100 microscope (Olympus, Tokyo, Japan).

### Colony-formation assay

The clonogenic potential of transfected or infected cells was evaluated by plate colony-formation assay. Cells were seeded onto plastic 6-well plates at an initial density of 1 × 10^3^ cells/well in appropriate growth media and incubated for 2 weeks. The cells were fixed with 4% paraformaldehyde, and stained with Crystal Violet Staining solution (Beyotime; C0121). The stained colonies were then counted and representative images were captured under an inverted microscope (Olympus Corporation; CX31, Tokyo, Japan).

### Wound healing assays

For the wound healing assays, the cells were cultured in 12-well plates and grown to 100% confluence. The cell monolayer was scratched with a 10 μL pipette tip to create a wound. Representative images of cell migration were taken using an optical microscope system.

### Transwell migration assays

The transwell chamber assay was performed to evaluate the migration ability of PCa cell lines using the 8-μm pore-size transwell plates (Corning, NY, USA). Briefly, 5 × 10^4^ cells were seeded on the upper chamber in 200 μL serum-free medium while the bottom chambers were filled with 500 μL medium containing 10% FBS (as chemoattractant). After incubation for 36 h, the cells on the upper surface of membranes were removed by a cotton swab, and the invaded cells on the lower surface of the upper chamber were stained with Crystal Violet Staining solution (Beyotime; C0121) at room temperature for 30 min. For visualization, the invaded cells were photographed (Olympus Corporation; CX31, Tokyo, Japan).

### *Fluorescence *in situ* hybridization (FISH)*

Cy3-labeled circSPON2 and FITC-labeled miR-331-3p probes were synthesized by GenePharma (Shanghai; China). The Fluorescent in Situ Hybridization Kit (Guangzhou Geneseed Biotech Co., Ltd., China) was used to hybridize the probes to cells. Images were captured using a Leica TCS-SP2-AOBS-MP confocal microscope (Leica Microsystem, Heidelberg, Germany). The probes used for FISH were as follows:

- circSPON2 probe: 5’-(Cy3)-CGCGGCCCTTGTGAGCAACAGAGATA-(Cy3)-3’;
- miR-331-3p probe: 5’-(FITC)-TTCTAGGATAGGCCCAGGGG-(FITC)-3’.

### RNA extraction and quantitative real-time PCR (qRT-PCR)

Total RNA extraction from cells/tissues was performed by using Trizol reagent (Life technologies), and measured by a NanoDrop 2000 spectrophotometer (Thermo Fisher Scientific, USA). For circRNA and mRNA, reverse transcriptions were performed using the PrimeScript RT Master Mix (Takara; RR036A). For miRNA, reverse transcriptions were performed using the PrimeScript™ RT Reagent Kit (Takara; RR037A) with specific stem-loop primers (CenePharma; China). Real-time PCR analyses were performed using the ChamQ SYBR qPCR Master Mix (Vazyme; Q311-02) on a Roter Gene 3000 sequence detection system (Corbett Research, Australia). The relative expression of circRNA, miRNA, and mRNA was calculated via the 2^−ΔΔCt^ method and standardized to endogenous GAPDH or U6. The primers used for real-time PCR are listed in Supplementary Table S3.

### Protein extraction and Western blot

Cells were collected and washed twice in 1 × PBS, then lysed in an ice-cold lysis buffer (Beyotime; P0013). For Western blot analysis, protein samples were mixed with loading buffer (Tanon; 180-8210D), separated by sodium dodecyl sulfate–polyacrylamide gel electrophoresis (SDS-PAGE), and then electroblotted onto polyvinylidene difluoride (PVDF; Millipore) membranes. After blocking in 5% nonfat-milk, the membranes were incubated with primary immunoblotting antibodies at 4 °C overnight and then incubated with horseradish peroxidase (HRP)-conjugated secondary antibody (Cell Signaling Technology, USA). Finally, the blots were visualized using a Chemistar™ High-sig ECL Western Blotting Substrate kit (Tanon; 180–501).

### Luciferase reporter assays

The promoter activity reporter vectors (pGL3.0-CAMK2N1 promoter) and PRMT5-overexpressing plasmid (pcDNA3.1-PRMT5) were co-transfected into PCa cells using Lipofectamine 3000 (Life Technologies). The sequences of circSPON2 and PRMT5-3’UTR and their corresponding mutation were designed, synthesized, and inserted into luciferase reporter vector, termed circSPON2-WT, circSPON2-Mut, PRMT5-3’-UTR-WT, and PRMT5-3’-UTR-Mut, respectively. These plasmids were individually co-transfected with miR-331-3p mimics or inhibitor into PCa cells, and the relative luciferase activity was examined by Dual Luciferase Assay Kit (Promega; USA).

### RNA pulldown assay

CircRNA pulldown was performed using biotin-labeled circSPON2 probes (CenePharma; China). Briefly, PCa cells were lysed in lysis buffer (Beyotime; P0013) Biotin-labeled circSPON2 probes or the negative control probes were added to the precleared lysate, and the mixtures were incubated at 4 °C overnight. Next, the mixture was incubated with 40 ~ 50 μL Streptavidin MagBeads (Genescript; L00424) for 1 h at 4 °C. The beads-probe-protein complex was washed four times with lysis buffer, and the total RNA bound to the beads was extracted by Trizol reagent (Life technologies), followed by reverse transcription and qRT-PCR analyses of circSPON2 and miR-331-3p.

### Chromatin immunoprecipitation (ChIP)

Cells (5 × 10^6^ cells per assay) were cross-linked with 1% formaldehyde (Sigma; F8775), quenched with 0.125 M glycine, and then sonicated using a Bioruptor UCD-300 (Diagenode) to yield chromatin fragments. Then 100 μg of lysate was immunoprecipitated with anti-IgG (negative control), anti-PRMT5 (Sigma; P0493), anti-H4R3me2s (Abcam: ab5823), or anti-H3R8me2s (Abcam; ab272149) antibodies immobilized on protein A-Sepharose beads (Sigma; P3391). The immunoprecipitated complexes were washed sequentially with washing buffer (10 mM Tris–HCl, 500 mM NaCl, 1% Trition X-100, 0.1% SDS, 0.5% Na-deoxycholate). After digestion with proteinase K (Beyotime; ST532), the DNA fragments were extracted with phenol:chloroform:isoamyl alcohol (25:24:1 v:v:v) and precipitated with 100% ethanol at -20 °C in the presence of 0.3 M sodium acetate (pH 5.2) and 2 µg of glycogen (Beyotime; D0812). Finally, the glycogen-protein pellet was suspended in H_2_O and the purified DNA was subjected to real-time PCR analyses. The ChIP primer sequences are listed in Supplementary Table S4.

### Hematoxylin and eosin (H&E) staining

Paraffin embedded tumor tissues were sectioned (4 μm) and dewaxed using xylene and rehydrated through a serial alcohol gradient. After washing with 1 × PBS, the slides were stained with hematoxylin and eosin (H&E; Beyotime; C0105) and dehydrated through increasing concentrations of ethanol and xylene.

### Immunohistochemistry (IHC) staining and scoring

Human PCa specimens and xenograft tissues were fixed in 10% neutral-buffered formalin solution (Sigma; HT501128), embedded in paraffin and then sectioned with a microtome (Leitz 1512, Germany). The sections were deparaffinized in xylene (Apollo; OR800028) and rehydrated in a descending ethanol (Aladdin; E111992) gradient. Antigen retrieval was performed in 10 mM citric acid buffer (Sigma; 27,487, pH 6.0) and then incubated with PRMT5 (Sigma; P0493) or Ki-67 (Abcam; ab15580) antibodies. After the slides had been rinsed, they were incubated with goat anti-rabbit horseradish peroxidase (HRP)-conjugated secondary antibodies (CST; #8114) and visualized in SignalStain® DAB Substrate solution (CST; #8059). The standard H‑score (scale of 0–300) was calculated according to the formula: H-score = ΣPi (i + 1), where “i” represents an intensity score and “Pi” is the percentage of immunostained cells.

### Xenografts model and *in vivo *imaging

Male BALB/c nude mice (6 ~ 8 weeks old) were purchased from the Model Animal Research Center of Nanjing University. For xenograft models, 5 × 10^6^ PCa cells were suspended in 100 μL PBS containing 15% Matrigel (BD) and subcutaneously injected into the flank of BALB/c nude mice. During the 30-day observation, mice were periodically examined for tumor growth, and tumor size was measured with digital calipers. The tumor volumes were calculated by the formula: volume (cm^3^) = 0.5 × length × width^2^. 30 days later, tumors were excised, weighed, and fixed in 4% paraformaldehyde for paraffin embedding.

To investigate tumor metastasis, 2 × 10^6^ PC-3 cells suspended in 100 μL PBS were injected into the tail vein of male BALB/c nude mice. The IVIS Spectrum animal imaging system (PerkinElmer) was used to check the metastatic loci formed by PC-3 cells in lung (40 days) with 100 μL XenoLight D-luciferin Potassium Salt (15 mg/mL; Perkin Elmer) per mouse. Mice were anesthetized and then sacrificed for tumors and metastases, which were further analyzed by H&E staining. All animal experiments and manipulations were reviewed and approved by the Experimental Animal Ethical Committee of Nanjing Medical University.

### Statistical analysis

Data were statistically analyzed using GraphPad Prism 8 software (Graphpad, San Diego, CA, USA) and SPSS 20.0 (IBM, SPSS, Chicago, IL, USA). Experimental data are expressed as mean ± standard deviation (SD), derived from triplicate samples of at least three independent experiments. Student’s *t*-test (unpaired, two tailed) was used to calculate statistical significance. A *P* value of less than 0.05 (*P < 0.05) was considered statistically significant.

## Results

### Upregulation of PRMT5 is associated with poor progression-free survival and the activation of multiple signaling pathways in PCa

**Fig. 1:**
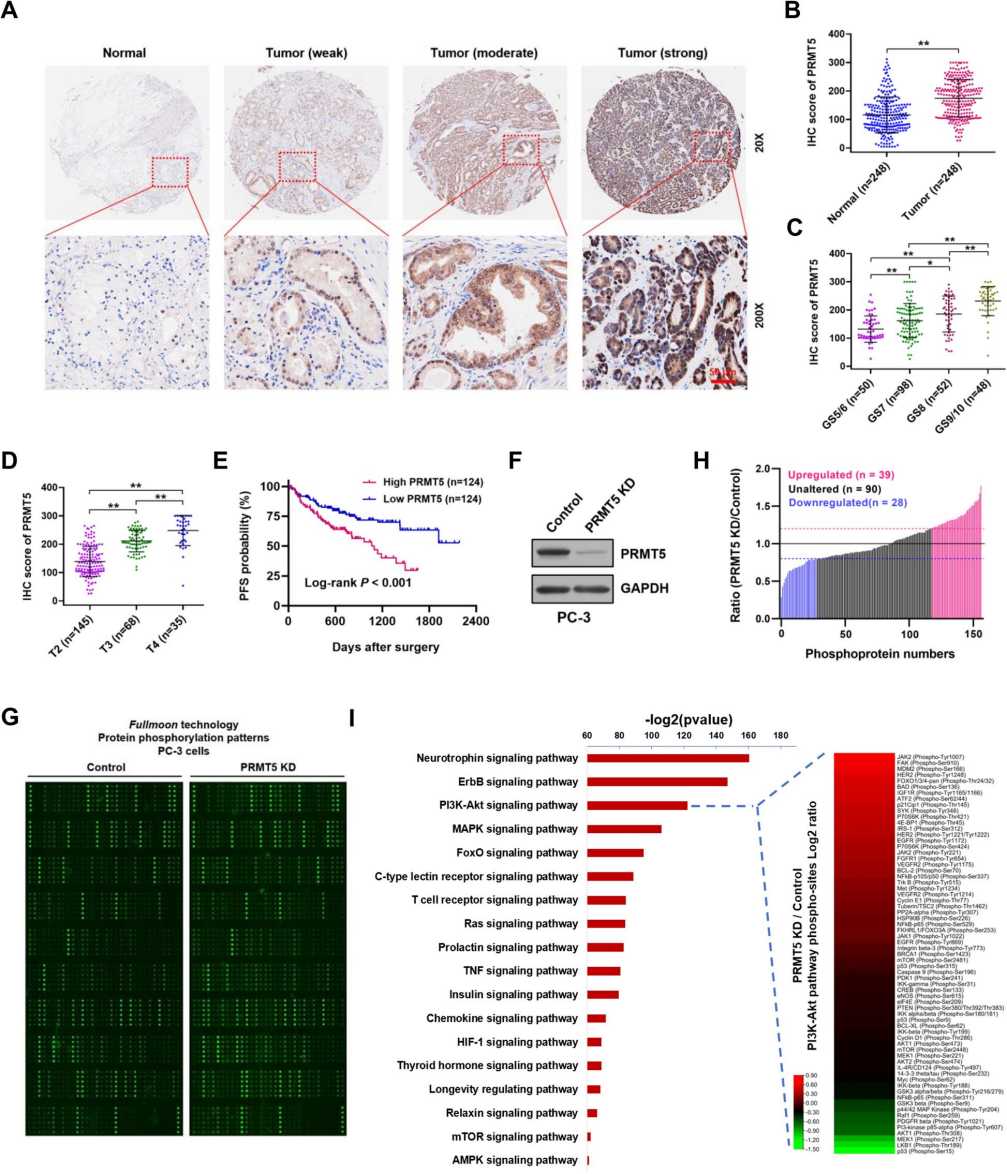
PRMT5 is upregulated in PCa tissues and activates multiple signaling pathways. **A** Representative images of IHC staining of PRMT5 in PCa patients and their adjacent normal tissues. Scale bar: 50 μm. **B** Relative expression of PRMT5 levels (IHC score) in PCa tumor and paired adjacent normal tissues (*n* = 248). **C** and **D** Comparison of PRMT5 levels (H score) in different Gleason scores (GS) and T stages were analyzed in PCa samples (*n* = 248). **E** Kaplan–Meier curve was used to evaluate the progression-free survival (PFS) of PCa patients with high (*n* = 124) or low (*n* = 124) PRMT5 expression (Log-rank, *P* < 0.001). Data are shown as mean ± SD. *P < 0.05, **P < 0.01. **F** siRNA-mediated PRMT5 knockdown in PC-3 cells was confirmed by Western blot, and GAPDH was used as an internal control. **G** Phosphorylation of all screened proteins from PRMT5-depleted and control PC-3 cells based on a phosphospecific protein microarray analysis. **H** Among a total of 157 phosphorylation sites designed in the antibody array, phosphorylation at 67 sites was significantly altered (39 increased and 28 decreased) in PRMT5-depleted sample, compared with the control (fold-change PRMT5 KD *vs*. Control ≥ 1.2). **I** KEGG analysis of proteins in the phospho-specific protein microarray according to biological process, cellular component, molecular function. (Right panel) Heat map showing the fold change in expression of phosphorylated proteins in the PI3K-Akt pathway based on the phospho-specific protein microarray

An increasing number of studies have shown that PRMT5, as an oncogene, plays an indispensable regulatory role in the pathological progression of several human malignancies [22]. We compared PRMT5 protein expression level between PCa and paired adjacent normal tissues via IHC staining. As shown in Fig. 1A, PRMT5 protein was preferentially located in the nuclei of tumor cells, with a small proportion in the cytoplasm shown by diffused or non-granular staining pattern. H score evaluation indicated that the protein levels of PRMT5 in PCa tissues were significantly higher than that in adjacent normal tissues (Fig. 1B), and elevated PRMT5 levels were associated with higher Gleason scores, higher T stages and shorter progression-free survival (PFS) (Fig. 1C-E; Supplementary Table S5).

To better understand the function of PRMT5 in PCa, we used a phospho-specific protein microarray containing 157 phosphoprotein antibodies to characterize the difference in phosphorylation patterns between PRMT5 knock down (PRMT5 KD) and control PC-3 cells. As shown in Fig. 1F, siRNA-mediated PRMT5 knockdown (PRMT5 KD) was confirmed by Western blot. Strikingly, the PRMT5 KD group exhibited 67 phosphorylation sites that significantly changed (39 phosphorylation sites significantly increased and 28 phosphorylation sites decreased compared to the control group) (Fig. 1G and H; fold change PRMT5 KD *vs*. Control ≥ 1.2). KEGG analysis revealed that proteins with significantly altered phosphorylation status were highly enriched in categories including ErbB, PI3K-Akt, MAPK, AMPK, JAK-STAT, mTOR, cell cycle, and NF-κB signaling pathways (Fig. 1I). These findings are consistent with previous reports that PRMT5 activates multiple signaling pathways, especially the PI3K-Akt signaling pathway, to facilitate tumorigenesis and cancer progression [23–27].

### CAMK2N1 is a transcriptional target of PRMT5 in PCa cells

**Fig. 2:**
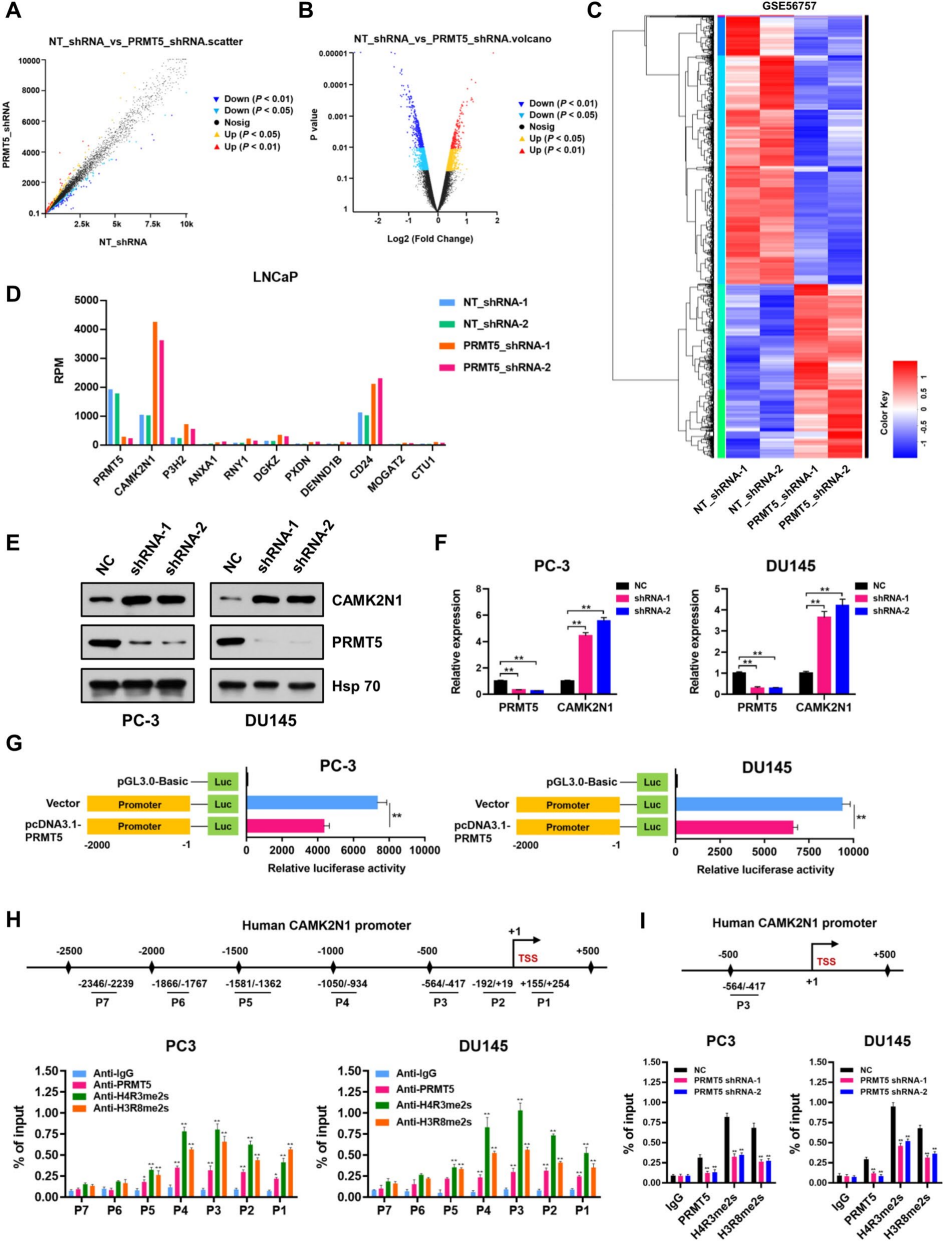
CAMK2N1 is a transcriptional target of PRMT5 in PCa cells. **A** Scatter plot of differentially expressed genes. **B** Volcano plot of differentially expressed genes. **C** Heatmap analyzed from RNA-seq data (GEO: GSE56757) listed the differentially expressed genes in LNCaP cells treated with negative control (NT-shRNAs) or PRMT5 shRNAs. **D** The most 10 upregulated genes in LNCaP cells treated with negative control (NT-shRNAs) or PRMT5 shRNAs analyzed from RNA-seq data (GEO: GSE56757). **E** Relative protein levels of CAMK2N1 were detected by Western blot in negative control (NC) or PRMT5-depleted (shRNAs) PC-3 and DU145 cells. **F** Relative mRNA levels of CAMK2N1 were detected by qRT-PCR assays in negative control (NC) or PRMT5-depleted (shRNAs) PC-3 and DU145 cells. **G** Promoter-driven luciferase reporter assays in PC-3 and DU145 cells transfected with pGL3.0 luciferase reporter plasmid containing promoter region of CAMK2N1 (-2000 to -1 bp) and pcDNA3.1-PRMT5, compared with that of cells co-transfected with pGL3.0-promoter (-2000 to -1 bp) and empty vector. **P < 0.01. **H** Relative enrichment of PRMT5 and its catalytic histone marks H4R3me2s and H3R8me2s on the promoter region of CAMK2N1 gene was evaluated by ChIP-qPCR assays in PC-3 and DU145 cells. P1 (+ 155 ~  + 254), P2 (-192 ~  + 19), P3 (-564 ~ -417), P4 (-1050 ~ -934), P5 (-1581 ~ -1362), P6 (-1866 ~ -1767) and P7 (-2346 ~ -2239). IgG was used as a negative control. **I** Relative enrichment of PRMT5 and its catalytic histone marks H4R3me2s and H3R8me2s at the selected loci (P3: -564 ~ -417) of CAMK2N1 promoter was evaluated by ChIP-qPCR assays in control (NC) or PRMT5-depleted PC-3 and DU145 cells. IgG was used as a negative control. Data were showed as mean ± SD. **P < 0.01

PRMT5, a known epigenetic regulator, governs the transcription of pivotal tumor suppressor genes by catalyzing histone H4R3 or H3R8 symmetric di-methylation (H4R3me2s or H3R8me2s). Both H4R3me2s and H3R8me2s have been extensively established as repressive histone marks in transcriptional regulation. We hypothesize that PRMT5 inhibits the transcription of some important target genes to drive PCa progression. The GEO dataset (accessible via GSE56757) from expression profiling array was used to investigate the role of PRMT5 in PCa progression by analyzing PRMT5-depleted or control PCa cells (LNCaP). Based on the profiles of differentially expressed genes, selected targets with log2 (fold change) > 1 or < -1, and *P* < 0.05, were illustrated by scatter plot (Fig. 2A), volcano plot (Fig. 2B), and heatmap of clustering analysis (Fig. 2C). The top 10 most upregulated target genes in PRMT5-depleted LNCaP cells from the dataset (GSE56757) are presented in Fig. 2D, which includes CAMK2N1, P3H2, ANXA1, RNY1, DGKZ, PXDN, DENND1B, CD24, MOGAT2, and CTU1. Among these candidate targets, calcium/calmodulin dependent protein kinase II inhibitor 1 (CAMK2N1) was the most upregulated gene in PRMT5-depleted LNCaP cells from the dataset. Western blot and qRT-PCR assays were performed to evaluate the expression levels of CAMK2N1 in PRMT5-depleted PC-3 and DU145 cells, respectively. Compared to those in control (NC) cells, the protein and mRNA levels of CAMK2N1 were dramatically increased in PRMT5-deficient PC-3 and DU145 cells (Fig. 2E and F), suggesting that PRMT5 plays a potential role in the transcriptional control of CAMK2N1.

To dissect underlying regulatory mechanisms, CAMK2N1 promoter-driven dual luciferase reporter-gene assays were performed. Our results showed that luciferase activity was strikingly decreased when pcDNA3.1-PRMT5 and pGL3.0-CAMK2N1 promoter constructs were co-transfected into PC-3 and DU145 cells (Fig. 2G). Next, chromatin immunoprecipitation (ChIP-qPCR) assays were conducted to validate the regulatory function of PRMT5 in CAMK2N1 transcription. Seven pairs of primers were designed across the promoter region of CAMK2N1 gene, and we observed that PRMT5 was significantly enriched at the promoter regions and at the vicinity of the transcription start site of CAMK2N1 gene in PC-3 and DU145 cells, especially at the region of − 1000 bp to + 1 bp from the transcription start site (Fig. 2H). Since PRMT5 can catalyze histone H4R3 or H3R8 symmetric di-methylation (H4R3me2s or H3R8me2s), we investigated whether PRMT5-mediated H4R3me2s and H3R8me2s are distributed at the promoter region of CAMK2N1. Although ChIP-qPCR assays revealed that both H4R3me2s and H3R8me2s marks were preferentially enriched at the promoter loci of CAMK2N1, we observed a significantly decreased H4R3me2s and H3R8me2s occupancy at the selected loci (P3) upon PRMT5 knockdown by shRNAs (Fig. 2I). Given the fact that CAMK2N1 is a direct transcriptional target of PRMT5 in PCa, we want to explore the correlation between PRMT5 and CAMK2N1 in PCa tissues. Using qRT-PCR analysis, we found that CAMK2N1 expression was significantly down-regulated in PCa tissues compared to normal tissues (Supplementary Fig. S1A). Moreover, correlation analysis revealed that PRMT5 expression negatively correlated with CAMK2N1 expression in our own cohort of 248 PCa patients (Supplementary Fig. S1B; *r* = -0.4279, *P* < 0.01).

### PRMT5 promotes PCa progression through PRMT5-CAMK2N1 axis

**Fig. 3:**
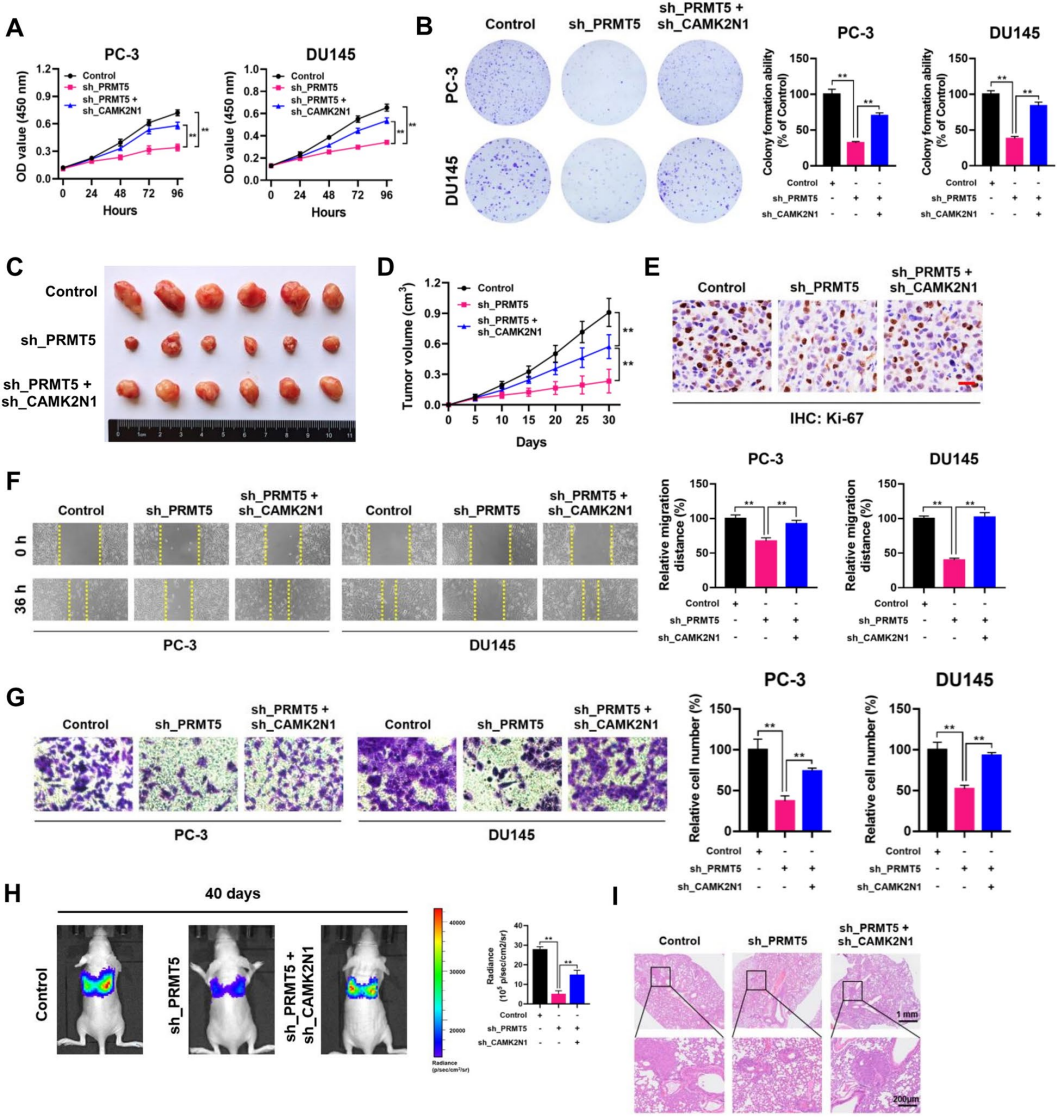
PRMT5 promotes PCa progression through the PRMT5-CAMK2N1 axis. **A** and **B** CCK-8 and plate colony-formation assays were performed to determine the proliferation abilities of PC-3 and DU145 cells treated with control, sh_PRMT5, or sh_PRMT5 + sh_CAMK2N1. **C** Image of subcutaneous xenografts in control, sh_PRMT5, sh_PRMT5 + sh_CAMK2N1 groups. **D** The tumor growth curves of xenografts were plotted in control, sh_PRMT5, sh_PRMT5 + sh_CAMK2N1 groups. **E** Representative IHC staining micrographs of Ki-67 in tumor xenografts were conducted. Scale bar = 20 μm. **F** and **G** Wound healing and transwell assays were performed to determine the migratory capabilities of PC-3 and DU145 cells treated with control, sh_PRMT5, sh_PRMT5 + sh_CAMK2N1. **H** BALB/c nude mice injected with cells (control, sh_PRMT5, sh_PRMT5 + sh_CAMK2N1; Five mice per group) via tail vein were imaged at 40 days by in vivo imaging system to evaluate the whole metastasis. **I** Representative images of H&E staining of lung metastasis loci. Data were showed as mean ± SD. **P < 0.01

To explore whether PRMT5 exerts its oncogenic role by modulating CAMK2N1 expression, a rescue assay between PRMT5 and CAMK2N1 was performed in PCa cells. We found that the expression of CAMK2N1 was largely abrogated by introducing CAMK2N1 shRNA (sh_CAMK2N1) in PRMT5-depleted PC-3 and DU145 cells (Supplementary Fig. S2). The CCK-8 and colony-formation assays (Fig. 3A and B) showed that silencing CAMK2N1 expression significantly reversed the PRMT5 knockdown-mediated proliferation defective phenotype in PC-3 and DU145 cells. In the xenograft tumor model, CAMK2N1 knockdown remarkably abolished the PRMT5 loss-induced growth inhibition of PC-3 xenografts (Fig. 3C-E). Wound-healing and transwell assays revealed that double-knockdown cells (sh_PRMT5 + sh_CAMK2N1) showed higher migratory capabilities than those of control cells (sh_PRMT5) (Fig. 3F and G). In the lung metastasis nude mouse model, suppression of CAMK2N1 led to increased metastasis of PRMT5-depleted PC-3 cells in the lungs (Fig. 3H). Furthermore, H&E staining revealed more metastatic foci in the sh_PRMT5 + sh_CAMK2N1 group than in the sh_PRMT5 group (Fig. 3I). These results demonstrated that PRMT5 may aggravate the aggressive behaviors of PCa cells, at least partially, through downregulating the expression of CAMK2N1.

### PRMT5 is a direct target gene of the tumor suppressor miR-331-3p

**Fig. 4:**
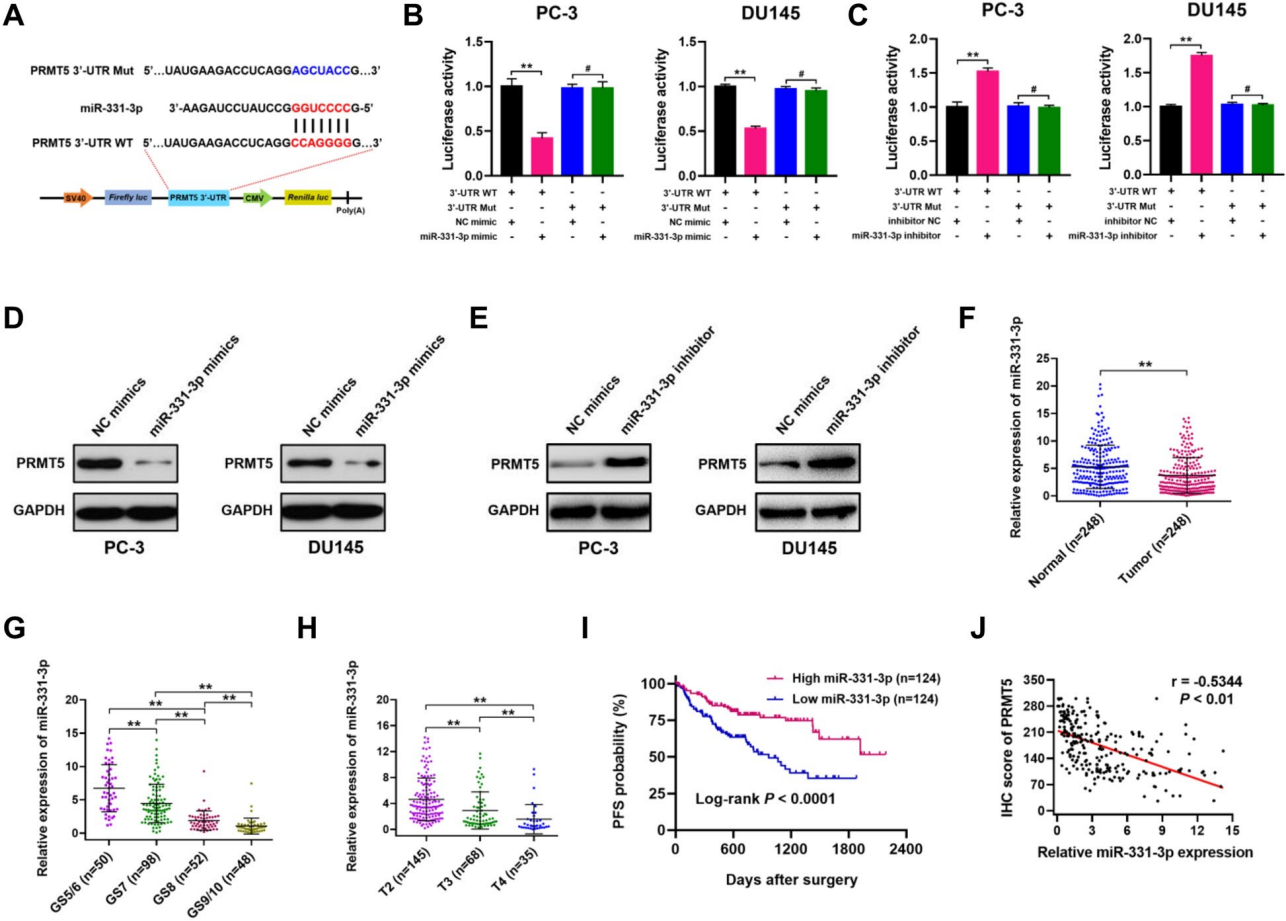
PRMT5 is a direct target gene of the tumor suppressor miR-331-3p. **A** Schematic illustration of PRMT5 3’-UTR wild-type (WT) and miR-331-3p binding site mutated (Mut) PRMT5 3’-UTR luciferases reporter vectors. **B** Relative luciferase activities were measured in PC-3 and DU145 cells transfected with PRMT5 3’-UTR WT or Mut luciferases reporter vectors and miR-331-3p or NC mimics. **C** Relative luciferase activities were measured in PC-3 and DU145 cells transfected with PRMT5 3’-UTR WT or Mut luciferases reporter vectors and miR-331-3p inhibitor or NC mimics. **D** PRMT5 protein expression was detected by Western blot in PC-3 and DU145 cells transfected with miR-331-3p or NC mimics. **E** PRMT5 protein expression was detected by Western blot in PC-3 and DU145 cells transfected with miR-331-3p inhibitor or NC mimics. **F** Relative expression of miR-331-3p levels in PCa tumor and paired adjacent normal tissues (*n* = 248) by qRT-PCR. **G** and **H** Comparison of miR-331-3p levels in different Gleason scores (GS) and T stages were analyzed in PCa samples (*n* = 248). **I** Kaplan–Meier curve was used to evaluate the progression-free survival (PFS) of PCa patients with high (*n* = 124) or low (*n* = 124) miR-331-3p expression (Log-rank, *P* < 0.0001). **J** The correlation analysis between the expression levels of miR-331-3p and PRMT5 in our own patient cohort (*n* = 248; *r* = -0.5344, *P* < 0.01)

Emerging studies have demonstrated that miRNAs exert important roles in driving the progression of PCa through modulating their targets [28, 29]. To identify the potential miRNAs targeting PRMT5, we performed bioinformatics analyses using TargetScan, mirDIP, miRTarBase, and miRDB databases. Among the potential miRNAs, miR-331-3p attracted more attention than other miRNAs, as it is one of the most downregulated miRNAs in PCa [30, 31]. To validate the direct interaction between miR-331-3p and PRMT5 3’-UTR, we performed dual luciferase reporter assays, where a reporter plasmid containing wild-type (WT) PRMT5 3’-UTR was co-transfected with miR-331-3p mimics in PC-3 and DU145 cells, which exhibited significantly decreased luciferase activity. In contrast, transfection of a reporter plasmid containing miR-331-3p binding site mutant (Mut) PRMT5 3’-UTR (CCAGGGG to AGCUACC) had no impact on cell luciferase activity (Fig. 4A and B). Co-transfection of the reporter plasmid containing wild-type (WT) PRMT5 3’-UTR with miR-331-3p inhibitor mimics resulted in increased luciferase activity, which was abolished when the miR-331-3p binding site of PRMT5 3’-UTR was mutated in PC-3 and DU145 cells (Fig. 4C). Next, the protein expression of PRMT5 was evaluated by Western blot in PC-3 and DU145 cells transfected with miR-331-3p or NC mimics. Our results showed that PRMT5 protein levels, but not mRNA levels, were downregulated in miR-331-3p-transfected PC-3 and DU145 cells (Fig. 4D; Supplementary Fig. S3A), thus leading to increased expression of CAMK2N1 (Supplementary Fig. S3B). In contrast, PRMT5 was obviously upregulated when miR-331-3p inhibitor was transfected into PC-3 and DU145 cells (Fig. 4E).

**Fig. 5:**
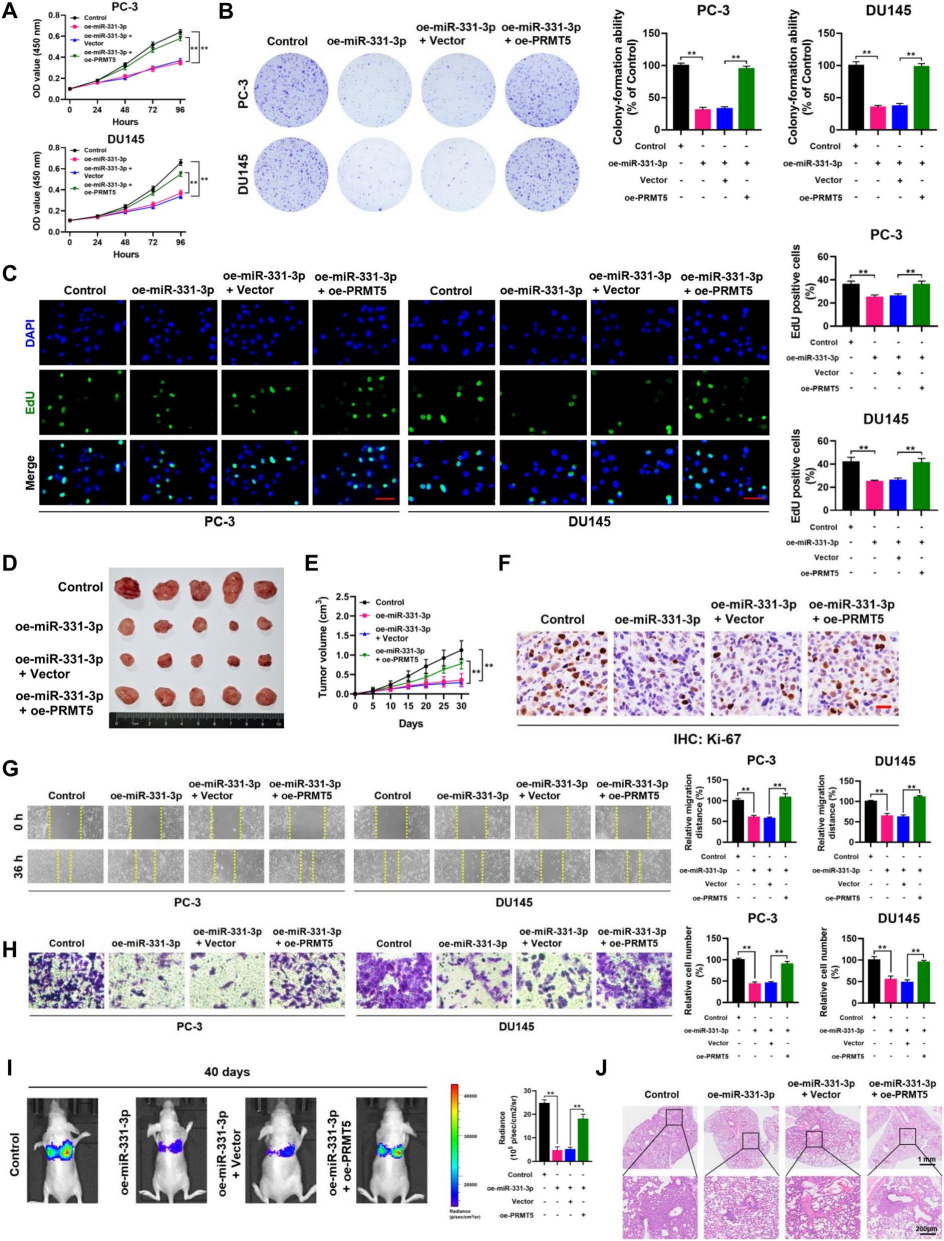
Restoration of PRMT5 expression reverses phenotype changes induced by miR-331-3p. **A**-**C** CCK-8, plate colony-formation and EdU incorporation assays were performed to determine the proliferation abilities of PC-3 and DU145 cells treated with control, oe-miR-331-3p, oe-miR-331-3p + Vector or oe-miR-331-3p + oe-PRMT5. **D** Image of subcutaneous tumor xenografts in control, oe-miR-331-3p, oe-miR-331-3p + Vector or oe-miR-331-3p + oe-PRMT5 groups. **E** The tumor growth curves of xenografts were plotted in control, oe-miR-331-3p, oe-miR-331-3p + Vector or oe-miR-331-3p + oe-PRMT5 groups. **F** Representative IHC staining micrographs of Ki-67 in tumor xenografts were conducted. Scale bar = 20 μm. **G** and **H** Wound healing and transwell assays were performed to determine the migratory capabilities of PC-3 and DU145 cells treated with control, oe-miR-331-3p, oe-miR-331-3p + Vector or oe-miR-331-3p + oe-PRMT5. **I** BALB/c nude mice injected with cells (control, oe-miR-331-3p, oe-miR-331-3p + Vector or oe-miR-331-3p + oe-PRMT5; Five mice per group) via tail vein were imaged at 40 days by in vivo imaging system to evaluate the whole metastasis. **J** Representative images of H&E staining of lung metastasis loci. Data were showed as mean ± SD. **P < 0.01

Moreover, by qRT-PCR analysis we found that miR-331-3p expression was down-regulated in PCa tissues compared to normal controls (Fig. 4F). The expression of miR-331-3p inversely correlated with higher Gleason scores and T stages (Fig. 4G and H; Supplementary Table S6). Kaplan–Meier analysis showed that PCa patients with lower miR-331-3p expression (Fig. 4I; Log-rank, *P* < 0.0001) were associated with poor progression-free survival (PFS). In addition, correlation analysis revealed that PRMT5 expression negatively correlated with miR-331-3p expression in our own cohort of 248 PCa patients (Fig. 5J; *r* = -0.5344, *P* < 0.01).

### Restoration of PRMT5 expression reverses phenotype changes induced by miR-331-3p

We further tested the effects of miR-331-3p on PCa cell proliferation and migration *in vitro* and *in vivo*. Restoration of PRMT5 expression in miR-331-3p-overexpressed PC-3 and DU145 cells was verified by qRT-PCR (Supplementary Fig. S4A). As shown in Fig. 5A-C, CCK-8, colony-formation, and EdU assays indicated that miR-331-3p overexpression (oe-miR-331-3p) suppressed the proliferation of PC-3 and DU145 cells. Rescue experiments demonstrated that PRMT5 overexpression reversed the inhibitory effect of miR-331-3p on the proliferation of PC-3 and DU145 cells. Notably, mouse xenograft model assays showed that restoration of PRMT5 expression significantly attenuated the inhibitory effect of miR-331-3p on the growth of PC-3 xenografts *in vivo* (Fig. 5D-F; Supplementary Fig. S4B). Wound-healing and transwell assays were used to investigate the migration ability. Our results suggest miR-331-3p overexpression significantly suppressed PC-3 and DU145 cell migration *in vitro*. Rescue experiments demonstrated that PRMT5 overexpression reversed the inhibitory effect of miR-331-3p on PC-3 and DU145 cell migration (Fig. 5G and H). The whole metastatic model was established and evaluated using an *in vivo* imaging system, and the results revealed a notable decrease of total metastatic sites in the miR-331-3p overexpressed (oe-miR-331-3p) group compared to that of control group on day 40 after injection. Restoration of PRMT5 expression dramatically reversed the inhibitory effect of miR-331-3p in metastatic foci formation (Fig. 5I), and isolated metastatic tissue sections were stained with H&E to validate the metastatic foci (Fig. 5J). To conclude, restoration of PRMT5 expression significantly reverses phenotype changes induced by miR-331-3p in PCa cells both *in vitro* and *in vivo*.

### Expression profiles of circRNAs and clinical features of circSPON2 in PCa

**Fig. 6:**
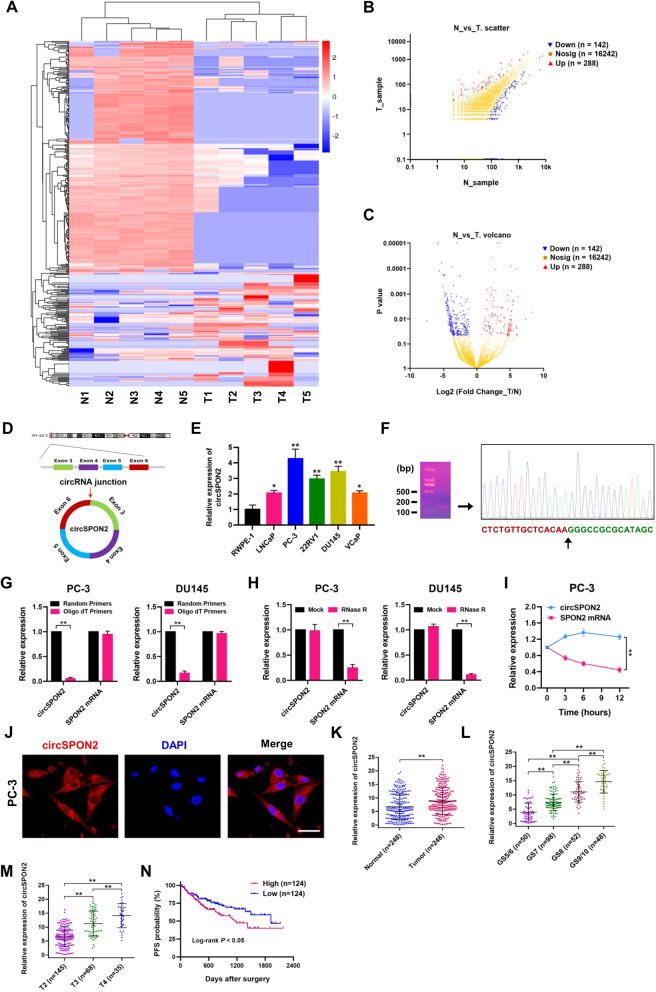
Expression profiles of circRNAs and clinical features of circSPON2 in PCa. **A** A cluster heap map presented the significantly dysregulated circRNAs in human PCa tissues relative to adjacent normal tissues. Red represents upregulation and green represents downregulation. **B** Scatter plot of differentially expressed circRNAs in PCa and adjacent normal tissues. The X- and Y-axes represent average signal values of circRNAs from PCa and adjacent normal tissues. **C** Volcano plot of differentially expressed circRNAs. The values of X- and Y-axes in the volcano plot are the fold change (log2 transformed) and *P* value between PCa and adjacent normal tissues, respectively. Red/Blue dots indicate twofold change differentially expressed genes with statistical significance (142 blue dots, downregulated circRNAs in PCa tissues, and 288 red dots, upregulated circRNAs in PCa tissues). Yellow dots indicate non-differentially expressed circRNAs in PCa tissues. **D** Schematic illustration of circSPON2 formation from SPON2 gene in chromosome 4. **E** Relative expression of circSPON2 was determined in human prostate epithelial cell line RWPE-1, and PCa cell lines (LNCaP, PC-3, 22RV1, DU145 and VCaP) by qRT-PCR. **F** The existence of circSPON2 was confirmed by RT-PCR and gel electrophoresis in PC-3 cells. The back splicing junction of circSPON2 was verified by Sanger sequencing. **G** qRT-PCR analysis for the circSPON2 and SPON2 mRNA using the template cDNA reverse-transcribed by random primers and oligo dT primers using RNAs from PC-3 and DU145 cells. **H** Stability of circSPON2 and SPON2 mRNA was assessed by RNase R treatment followed by qRT-PCR. **I** qRT-PCR assay for the expression of circSPON2 and SPON2 mRNA in PC-3 cells treated with Actinomycin D (2 μg/mL) at the indicated time points. **J** Fluorescence in situ hybridization (FISH) with Cy3-labeled circSPON2 probes (red) were performed to detect the location of circSPON2 in PC-3 cells. Scale bar = 20 μm. **K** Comparison of circSPON2 levels between PCa and paired adjacent normal tissues (*n* = 248). **L** Comparison of circSPON2 levels in different Gleason scores (GS) were analyzed in PCa tissues (*n* = 248). **M** Comparison of circSPON2 levels in different T stages were analyzed in PCa tissues (*n* = 248). **N** Kaplan–Meier curve was used to evaluate the progression-free survival (PFS) of PCa patients with high (*n* = 124) or low (*n* = 124) circSPON2 expression (Log-rank, *P* < 0.05). Data were showed as mean ± SD. *P < 0.05, **P < 0.01

Recent studies have shown that circRNAs are vital regulators in various cancers. To explore the expression profiles of circRNAs in PCa, we compared five pairs of PCa tissues and adjacent noncancerous tissues using circRNA RNA-seq analyses. Out of a total of 16,672 circRNAs identified in PCa tissues, 430 circRNAs were differentially expressed between PCa and paired normal prostate tissues (Supplementary Table S7). These circRNAs and their host genes were found to be in diverse genomic regions (Supplementary Fig. S5A). The chromosomal distribution and expression of identified circRNAs are shown by Circos plot (Supplementary Fig. S5B). Next, the composition of significantly expressed circRNAs was analyzed to identify their genomic origin. Our results showed that about 92.32% (4590/4972) of circRNAs were generated from exons (Supplementary Fig. S5C). Differentially expressed circRNAs are shown by hierarchical clustering heatmap (Fig. 6A). As illustrated in the scatter and volcano plots (Fig. 6B and C), 288 circRNAs were significantly upregulated in PCa tissues, whereas 142 circRNAs were significantly downregulated based on log2 (Fold change) ≥ 1 or ≤ -1, *P* < 0.05, and FDR < 0.05. In this study, we focused on the most upregulated circRNAs and matched them with circBase, and further studied the most upregulated circRNA determined in our RNA-seq analyses, Hsa_circ_0068835 (termed circSPON2 in the remainder of the article).

CircSPON2 is derived from the SPON2 gene on chromosome 4, as a product of back-splicing of exon 3, 4, 5, and 6 (1315 bp) (Fig. 6D). In addition, we also compared circSPON2 expression levels between PCa cell lines (PC-3, 22RV1, DU145, VCaP, and LNCaP) and the normal prostate epithelial cell line (RWPE-1). Compared to RWPE-1, PCa cells had profoundly elevated expression of circSPON2 (Fig. 6E). PCR and agarose gel electrophoresis assays confirmed that divergent primers could amplify the circSPON2 from reverse-transcribed RNA (cDNA), and the presence of the back-splicing junction of circSPON2 was confirmed by Sanger sequencing (Fig. 6F). Reduction in reverse-transcription efficiency by oligo dT primers due to the lack of poly (A) tail also demonstrated the circularity of circSPON2 (Fig. 6G). Furthermore, circSPON2 was more resistant to RNase R treatment, while SPON2 mRNA was significantly degraded by this treatment in PC-3 and DU145 cells (Fig. 6H). Figure 6I shows that circSPON2 was much more stable than SPON2 mRNA after Actinomycin D (a transcription inhibitor) treatment in PC-3 cells. To investigate the subcellular localization of circSPON2 in PCa cells, fluorescence in situ hybridization (FISH) assays were performed using Cy3-labeled circSPON2. Our results clearly showed that circSPON2 was predominately enriched in the cytoplasm of PC-3 cells (Fig. 6J), indicating that circSPON2 may function in the cytoplasm of PCa cells.

To investigate the expression pattern of circSPON2 in PCa tissues, relative expression levels of circSPON2 in PCa tissues and paired normal tissues were evaluated by quantitative real-time PCR (qRT-PCR) assays. In a cohort of 248 PCa patients, increased circSPON2 expression levels were observed in 68.6% (170 out of 248) PCa tissues compared to normal controls (Fig. 6K). The expression of circSPON2 was found to be increased with higher Gleason scores and T stages (Fig. 6L and M; Supplementary Table S8). Next, the PCa patients were divided into high- (*n* = 124) and low- expression (*n* = 124) groups based on the median circSPON2 expression level. Kaplan–Meier analysis showed that patients with high circSPON2 expression were associated with lower progression-free survival (PFS) (Fig. 6N; Log-rank). Taken together, our data indicate that upregulation of circSPON2 is closely associated with unfavorable clinical outcomes of PCa patients.

### CircSPON2 promotes PCa cell proliferation and migration

**Fig. 7:**
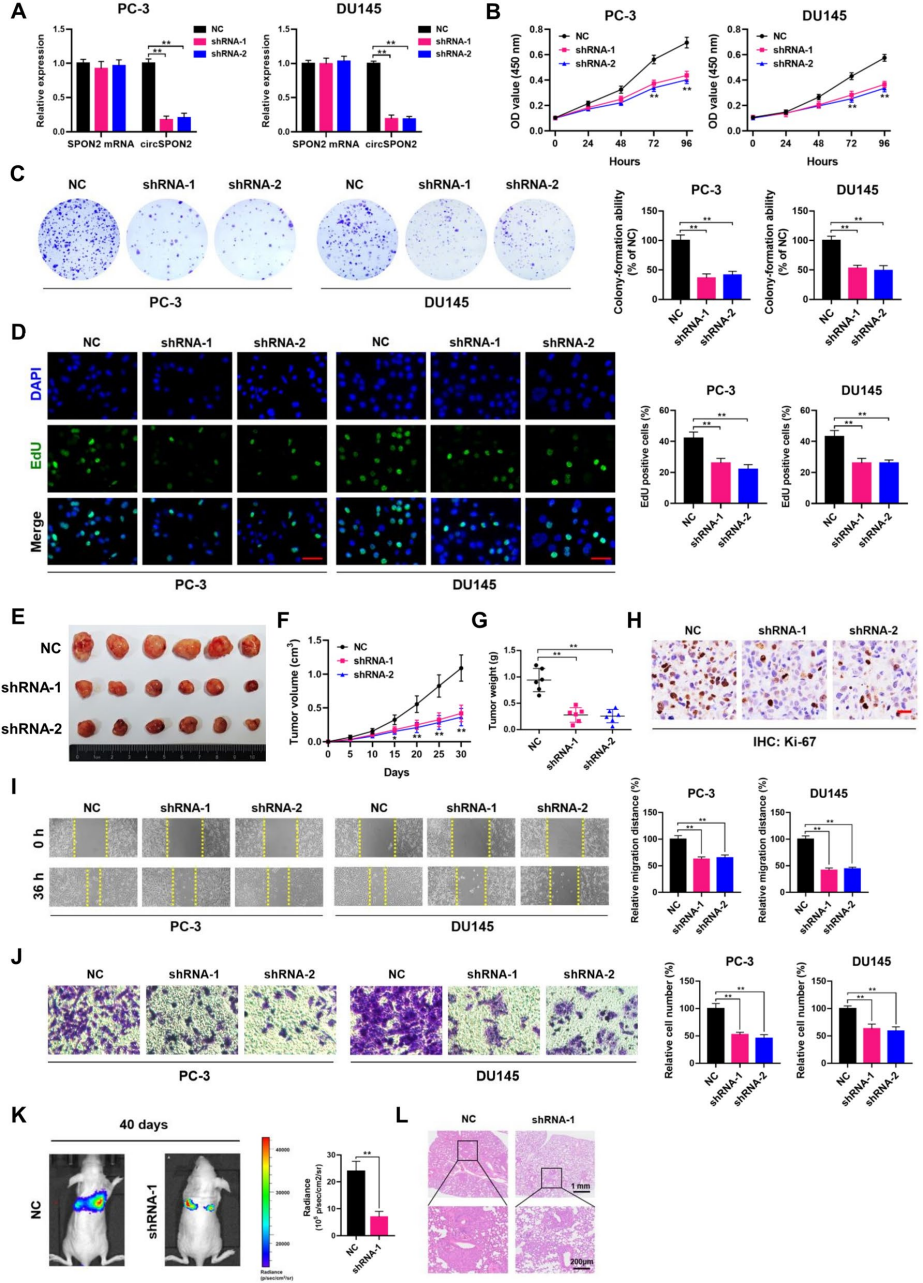
CircSPON2 promotes PCa cell proliferation and migration. **A** qRT-PCR analysis of circSPON2 and SPON2 mRNA in PC-3 and DU145 cells treated with negative control (NC) or circSPON2 shRNAs. **B**-**D** CCK-8, plate colony-formation and EdU incorporation assays were performed to determine the proliferation abilities of PC-3 and DU145 cells treated with negative control (NC) or circSPON2 shRNAs. **E** Image of subcutaneous tumor xenografts in negative control (NC) or circSPON2 shRNAs groups. **F** The tumor growth curves of xenografts were plotted in negative control (NC) or circSPON2 shRNAs groups. **G** The tumor weights of xenografts were evaluated. **H** Representative IHC staining micrographs of Ki-67 in tumor xenografts were conducted. Scale bar = 20 μm. **I** and **J** Wound healing and transwell assays were performed to determine the migratory capabilities of PC-3 and DU145 cells treated with negative control (NC) or circSPON2 shRNAs. **K** BALB/c nude mice injected with cells (NC and shRNA-1; five mice per group) via tail vein were imaged at day 40 by in vivo imaging system to evaluate the whole body metastasis. **L** Representative images of H&E staining of lung metastasis loci. Data were showed as mean ± SD. **P < 0.01

To evaluate the biological roles of circSPON2 in PCa, two short hairpin RNAs (shRNAs) that specifically target the back-splicing junction of circSPON2 were designed. The knockdown efficiency was confirmed by qRT-PCR analyses, which showed that both shRNAs result in > 80% reduction of circRNA abundance. As shown in Fig. 7A, the expression levels of circSPON2 were significantly decreased in shRNA-infected PC-3 and DU145 cells compared to negative control (NC) cells, without any alteration in the mRNA expression of the linear form of SPON2. CCK-8, colony formation, EdU incorporation assay demonstrated that downregulation of circSPON2 inhibited the proliferation of PC-3 and DU145 cells (Fig. 7B-D). In agreement with *in vitro* results, the shRNA lentivirus-mediated knockdown of circSPON2 remarkably inhibited the growth of PC-3 xenografts in vivo (Fig. 7E-G). Ki-67 staining clearly revealed that circSPON2 knockdown reduced the proliferation activity of PC-3 cells *in vivo* (Fig. 7H). IHC staining and qRT-PCR assays showed that PRMT5 was downregulated in circSPON2-depleted xenograft tissues, whereas CAMK2N1 was significantly increased after circSPON2 knockdown in PC-3 xenografts. (Supplementary Fig. S6A and B). Moreover, circSPON2 knockdown also suppressed the migratory and invasive capabilities of PC-3 and DU145 cells *in vitro* (Fig. 7I and J), and the formation of metastatic foci in the lungs of metastatic mouse models in vivo (Fig. 7K and L)*.*

To further investigate the function of circSPON2, a lentiviral circSPON2 overexpression vector was constructed (pLVX-circSPON2). As shown in Supplementary Fig. S7A, pLVX-circSPON2 significantly increased the expression levels of circSPON2 in PC-3 and DU145 cells. Overexpression of circSPON2 increased the proliferation capabilities of PC-3 and DU145 cells *in vitro* (Supplementary Fig. S7B-D), and promoted the growth of PC-3 xenografts *in vivo* (Supplementary Fig. S7E-H). Furthermore, results from wound-healing and transwell assays showed that the cell migration abilities were significantly enhanced in PC-3 and DU145 cells when circSPON2 was overexpressed, compared with those in control cells (Supplementary Fig. S7I and J).

### CircSPON2 serves as a sponge of tumor suppressor miR-331-3p in PCa

**Fig. 8:**
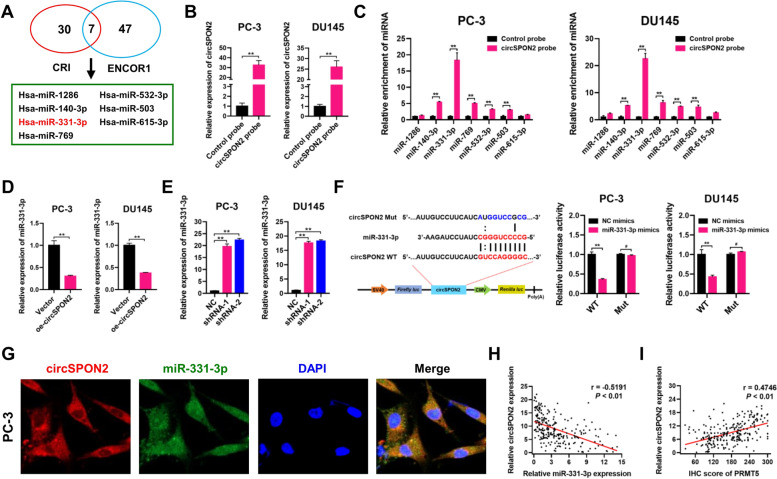
CircSPON2 serves as a sponge of tumor suppressor miR-331-3p in PCa. **A** Seven miRNAs were predicted as the potential targets of circSPON2 by CRI and ENCORI databases. **B** CircSPON2 in PC-3 and DU145 cells was pulled down by a circSPON2-specific probe and determined by qRT-PCR assays. **C** Relative levels of candidate miRNAs were determined by qRT-PCR assays after being pulled down by circSPON2-specific probe or control oligo probe. **D** Relative levels of miR-331-3p were detected by qRT-PCR assays in PC-3 and DU145 cells treated with vector or oe-circSPON2. **E** Relative levels of miR-331-3p were detected by qRT-PCR assays in PC-3 and DU145 cells treated with negative control (NC) or circSPON2 shRNAs. **F** Luciferase reporter assay of PC-3 and DU145 cells co-transfected with miR-331-3p mimics and luciferase reporter plasmid containing wild-type (WT) and miR-331-3p binding site mutated (Mut) circSPON2. **G** FISH with Cy3-labeled circSPON2 probes (red) and FITC-labeled miR-331-3p (green) were performed to detect the location of circSPON2 and miR-331-3p in PC-3 cells. **H** The correlation analysis between the expression levels of miR-331-3p and circSPON2 in our own patient cohort (*n* = 248; *r* = -0.5191, *P* < 0.01). **I** The correlation analysis between the expression levels of PRMT5 and circSPON2 in our own patient cohort (*n* = 248; *r* = 0.4746, *P* < 0.01). Data were showed as mean ± SD. *P < 0.05, **P < 0.01

Given the observation that circSPON2 is predominantly localized in the cytoplasm of PCa cells, we further explored whether circSPON2 can regulate the biological behaviors of PCa cells by sponging miRNAs. Downstream miRNAs that may be regulated by circSPON2 were predicted using the Circular RNA Interactome database and the Encyclopedia of RNA Interactomes database. Bioinformatics analyses revealed that seven miRNAs, including miR-1286, miR-140-3p, miR-331-3p, miR-769, miR-532-3p, miR-503, and miR-615-3p, were potential targets of circSPON2 (Fig. 8A). To validate the association between circSPON2 and the miRNAs, RNA *in vivo* immunoprecipitation (RIP) assays were executed using a biotin-labeled circSPON2 probe in PC-3 and DU145 cells. As shown in Fig. 8B, qRT-PCR assays showed that circSPON2 was specifically pulled down with the biotin-labeled probe, compared to the control oligo probe. Then, miRNAs were pulled down using biotin-labeled circSPON2 probe in PC-3 and DU145 cells. Interestingly, miR-331-3p was the most enriched miRNA among the seven miRNAs (Fig. 8C), indicating that miR-331-3p may be a potential circSPON2-associated miRNA in PCa cells. Moreover, the expression of miR-331-3p was significantly decreased when circSPON2 was overexpressed (Fig. 8D). Conversely, the expression of miR-331-3p was significantly increased in circSPON2-depleted PC-3 and DU145 cells (Fig. 8E). Taken together, our data indicate that miR-331-3p is a direct target of circSPON2 in PCa cells.

To further confirm the interaction between circSPON2 and miR-331-3p, luciferase reporter assays were performed. Commercially synthesized miR-331-3p or NC mimics were co-transfected with circSPON2 luciferase reporters into PC-3 and DU145 cells, respectively. Luciferase reporter activities were significantly suppressed by miR-331-3p mimics, compared with those using negative control mimics (Fig. 8F). Strikingly, the observed luciferase reporter activity repression was abolished when the binding sites of miR-331-3p were mutated (circSPON2 Mut). FISH using FITC-labeled miR-331-3p probes revealed that miR-331-3p was distributed in the cytoplasm and nucleus of PC-3 cells (Fig. 8G). In addition, correlation analysis revealed that circSPON2 expression negatively correlated with miR-331-3p expression (Fig. 8H; *r* = -0.5191, *P* < 0.01), whereas circSPON2 and PRMT5 expression levels (Fig. 8I; *r* = 0.4746, *P* < 0.01) exerted a positive correlation in our own cohort of 248 PCa patients.

### CircSPON2 promotes PCa progression by sponging miR-331-3p

**Fig. 9:**
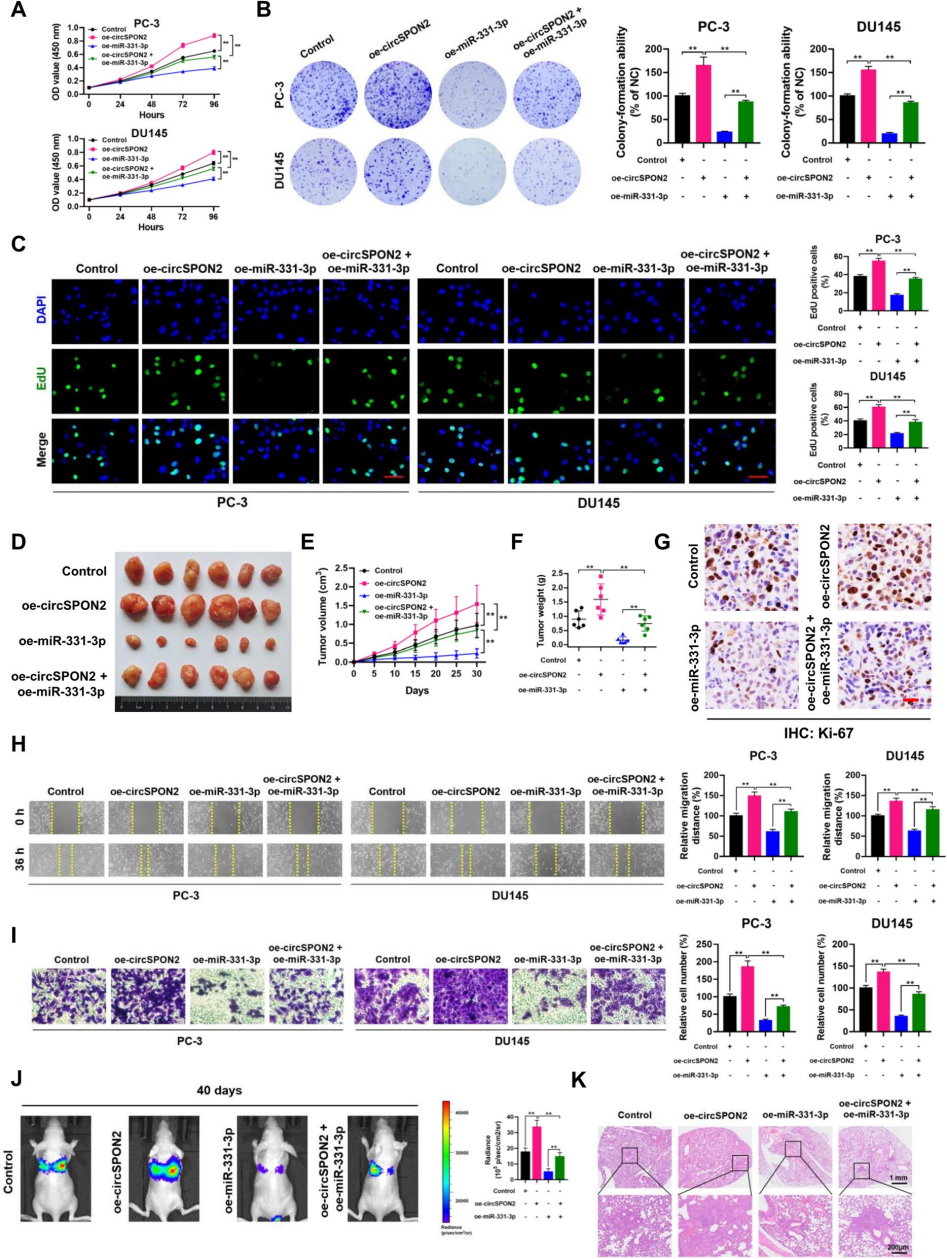
CircSPON2 promotes PCa progression by sponging miR-331-3p. **A**-**C** CCK-8, plate colony-formation and EdU incorporation assays were performed to determine the proliferation abilities of PC-3 and DU145 cells treated with control, oe-circSPON2, oe-miR-331-3p or oe-circSPON2 + oe-miR-331-3p. **D** Image of subcutaneous tumor xenografts in control, oe-circSPON2, oe-miR-331-3p or oe-circSPON2 + oe-miR-331-3p groups. **E** The tumor growth curves of xenografts were plotted in control, oe-circSPON2, oe-miR-331-3p or oe-circSPON2 + oe-miR-331-3p groups. **F** The tumor weights of xenografts were evaluated. **G** Representative IHC staining micrographs of Ki-67 in tumor xenografts were conducted. Scale bar = 20 μm. **H** and **I** Wound healing and transwell assays were performed to determine the migratory capabilities of PC-3 and DU145 cells treated with control, oe-circSPON2, oe-miR-331-3p or oe-circSPON2 + oe-miR-331-3p. **J** BALB/c nude mice injected with cells (control, oe-circSPON2, oe-miR-331-3p or oe-circSPON2 + oe-miR-331-3p; Five mice per group) via tail vein were imaged at 40 days by in vivo imaging system to evaluate the whole metastasis. **K** Representative images of H&E staining of lung metastasis loci. Data were showed as mean ± SD. **P < 0.01

To investigate whether circSPON2 promotes PCa progression by sponging miR-331-3p, rescue experiments were performed using the miR-331-3p mimic in circSPON2-overexpressed PC-3 and DU145 cells. Moreover, overexpression of miR-331-3p reversed the enhanced proliferative capacity caused by circSPON2 overexpression in PC-3 and DU145 cells (Fig. 9A-C). Similar effects were observed in PC-3 xenografts overexpressing circSPON2 *in vivo*, where the increased proliferative capability was attenuated by miR-331-3p (Fig. 9D-G). The expression levels of PRMT5 and CAMK2N1 were examined in these xenografts by IHC staining and qRT-PCR assays. As shown in Supplementary Fig. S8A and B, overexpression of circSPON2 upregulated the expression of PRMT5 and reduced CAMK2N1 expression in PC-3 xenografts. On the contrary, overexpression of miR-331-3p inhibited the expression of PRMT5, thus leading to increased CAMK2N1 expression. However, these effects were abolished if circSPON2 and miR-331-3p were co-overexpressed. Furthermore, wound healing and transwell assays suggested that increased circSPON2 expression promoted PC-3 and DU145 cell migration (Fig. 9H-I). However, miR-331-3p significantly reversed the enhanced migratory capabilities induced by circSPON2 overexpression, as shown by *in vivo* imaging analysis after 40 days and H&E staining (Fig. 9J and K). Taken together, these findings suggest that circSPON2 promotes PCa progression by sponging miR-331-3p.

## Discussion

PRMT5 is the major type II arginine methyltransferase that catalyzes the transfer of two methyl groups symmetrically to the arginine residues of either histone or non-histone proteins. In this study, epigenetic regulator PRMT5 was shown to inhibit the transcriptional output of CAMK2N1 by depositing repressive histone marks H4R3me2s and H3R8me2s on the proximal promoter region of CAMK2N1, thus resulting in malignant progression of PCa both *in vitro* and *in vivo*. Moreover, the expression of circSPON2, a candidate circRNA in PCa tissues identified by RNA-seq, was associated with poor clinical outcomes in PCa patients. Further results showed that circSPON2 induced PCa cell proliferation and migration, and that the circSPON2-induced effects were counteracted by miR-331-3p. Particularly, circSPON2 acted as a ceRNA of miR-331-3p to attenuate the repressive effects of miR-331-3p on its downstream target PRMT5.

CAMK2N1, characterized as an inhibitor of CaMKII (calcium/calmodulin-dependent protein kinase II) [32–35], has been identified as a downstream transcriptional target of PRMT5 in PCa cells. PRMT5-mediated H4R3me2s and H3R8me2s modifications were significantly enriched at the promoter regions and at the vicinity of the transcription start site of CAMK2N1 gene in PCa cells, especially at the core region of CAMK2N1 promoter. CAMK2N1 has been widely recognized as a bona fide tumor suppressor due to its crucial role in attenuating tumorigenesis and PCa progression [36, 37]. However, previous studies have mainly focused on the regulation of CAMK2N1 translation. For example, Wu and colleagues have reported that miR-129-5p contributes to docetaxel resistance in PCa by repressing CAMK2N1 expression [38]. So far, there has been no evidence that shows the association between arginine methylation at histones and the transcriptional inactivation of CAMK2N1. Therefore, it would be interesting to elucidate the mechanisms of arginine methylation at histones, and further analyze the cross-talks between arginine methylation marks and other histone modifications in the regulation of CAMK2N1 in PCa.

PRMT5 is known to repress the transcription of critical tumor suppressors by inducing histone H4R3 or H3R8 symmetric di-methylation at the promoter regions of targets (H4R3me2s or H3R8me2s), an event proposed as repressive histone modifications in transcriptional control [39, 40]. Our study shows for the first time that PRMT5 (also known as SKB1) is a potential target of miR-331-3p. PRMT5 has both histone and non-histone protein substrates, including histone 3 or 4, SWI/SNF, N-CoR/SMRT, COPR5, p53, MYC and CBP [4, 39, 41–43], and has been implicated in the regulation of diverse cellular events, such as cell differentiation, repair of DNA damage, ribosome assembly, angiogenesis, germ cell formation and RNA metabolism [22]. PRMT5, as an oncogene, plays an indispensable regulatory role in the pathological progression of several human malignancies, including lung [44], gastric [45], breast cancer [46], lymphoma [47], leukemia [48] and glioblastoma [49], by depositing symmetric di-methylation marks on the arginine residues of substrates. Our study discovered that miR-331-3p levels are negatively correlated with PRMT5 protein levels in our own cohort of PCa patients. More importantly, restoration of PRMT5 was observed to significantly reverse the miR-331-3p-induced phenotype changes of PCa cells *in vitro* or *in vivo*, indicating that circSPON2-miR-331-3p-mediated PRMT5 expression elevation may be an important driving force for PCa progression. Given the fact that miRNAs usually have multiple downstream targets, we could not rule out the possibility that miR-331-3p might also regulate PCa progression through other potential targets.

Recent studies have indicated that circRNAs are tightly associated with a wide range of physiological and pathological processes in human diseases, including cancer, diabetes, neurodegenerative diseases, atherosclerosis, cardiovascular diseases and inflammatory disorders [50]. CircRNAs have been shown to participate in tumorigenesis, progression, and metastasis of diverse human malignancies [51], and have been reported to control the malignant phenotype of PCa cells by serving as competitive endogenous RNA molecules. For example, circCSNK1G3 has been shown to promote the progression of PCa by interacting with miR-181 [52]. CircAGO2 exhibits oncogenic properties by activating human antigen R (HuR) protein to facilitate the formation of AGO2-miRNA complexes, which then leads to the abolishment of AGO2 binding, the inhibition of AGO2/miRNA-mediated gene silencing, and the promotion of tumorigenesis and progression [53]. Further, Yang and colleagues have reported that dysregulation of p53-RBM25-mediated circAMOTL1L biogenesis promotes epithelial-mesenchymal transition (EMT) and PCa progression through the circAMOTL1L-miR-193a-3p-PCDH regulatory axis [54].

In this study, we performed a comparative RNA-seq analysis to evaluate the expression profiles of circRNAs in PCa and adjacent normal tissues. CircSPON2, the most upregulated circRNA in our RNA-seq analyses, was chosen for further analyses. CircSPON2 is derived from exons 3, 4, 5 and 6 of its host gene SPON2 (also known as Mindin, DIL1, or M-Spondin), which encodes an extracellular matrix protein that binds to integrin receptors [55]. Through qRT-PCR analysis, circSPON2 was found to be upregulated in PCa tissues compared to paired normal controls. Importantly, elevation of circSPON2 expression correlated with higher Gleason scores and T stages and associated with poor progression-free survival (PFS), suggesting that circSPON2 plays pivotal biological roles in PCa. In agreement with this, further experiments revealed that depletion of circSPON2 attenuated the proliferative and metastatic capabilities of PCa cells *in vitro* and *in vivo*. These results suggest that circSPON2 is required for the aggressive phenotype exerted during the pathogenesis and development of PCa. To the best of our knowledge, for the first time, this study shows that circSPON2 exerts specific oncogenic roles in PCa.

Studies have shown that circRNAs play a role as sponges for miRNAs in modulating the expression of miRNA target genes in various cancers [20]. In this study, FISH results revealed that circSPON2 is preferentially distributed in the cytoplasm of PCa cells. Bioinformatics analyses, RNA pulldown, and luciferase reporter assays revealed that circSPON2 functions as a sponge of miR-331-3p in PCa cells. Furthermore, qRT-PCR analyses showed that downregulation of miR-331-3p is associated with worse clinical outcome in our cohort of PCa patients. To further confirm our observation, we overexpressed miR-331-3p in PCa cells and observed proliferation and metastasis defects in PCa cells *in vitro* or *in vivo*. Our findings are consistent with those observed in previous studies, which have shown that miR-331-3p is a tumor suppressor in a variety of human malignancies, including lung [56], colorectal [57], gastric [58], PCa [30], and melanoma [59]. We acknowledge that the main limitation to this study design is the inability to look deeply into the regulation of circSPON2 in PCa cells. In eukaryotic cells, many protein-coding genes generate linear mRNAs and circRNAs, but it is largely unknown how the circRNA is controlled or regulated. Liang et al. showed that inhibition or slowing of pre-mRNA processing events can profoundly increase the steady-state levels of circRNAs, in part by increasing their biogenesis [60]. Westholm et al. proposed that the global differences in circRNA levels that are observed across cell types may be due to differences in the concentrations or activities of pre-mRNA processing components [61]. Collectively, pre-mRNA processing events or components may be involved in the regulation of circRNAs. Moreover, mutant proteins, such as SF3b1 and U2AF1, may alter the amounts of linear or circular RNAs made from protein-coding genes, thereby resulting in disease phenotypes [62].

**Fig. 10:**
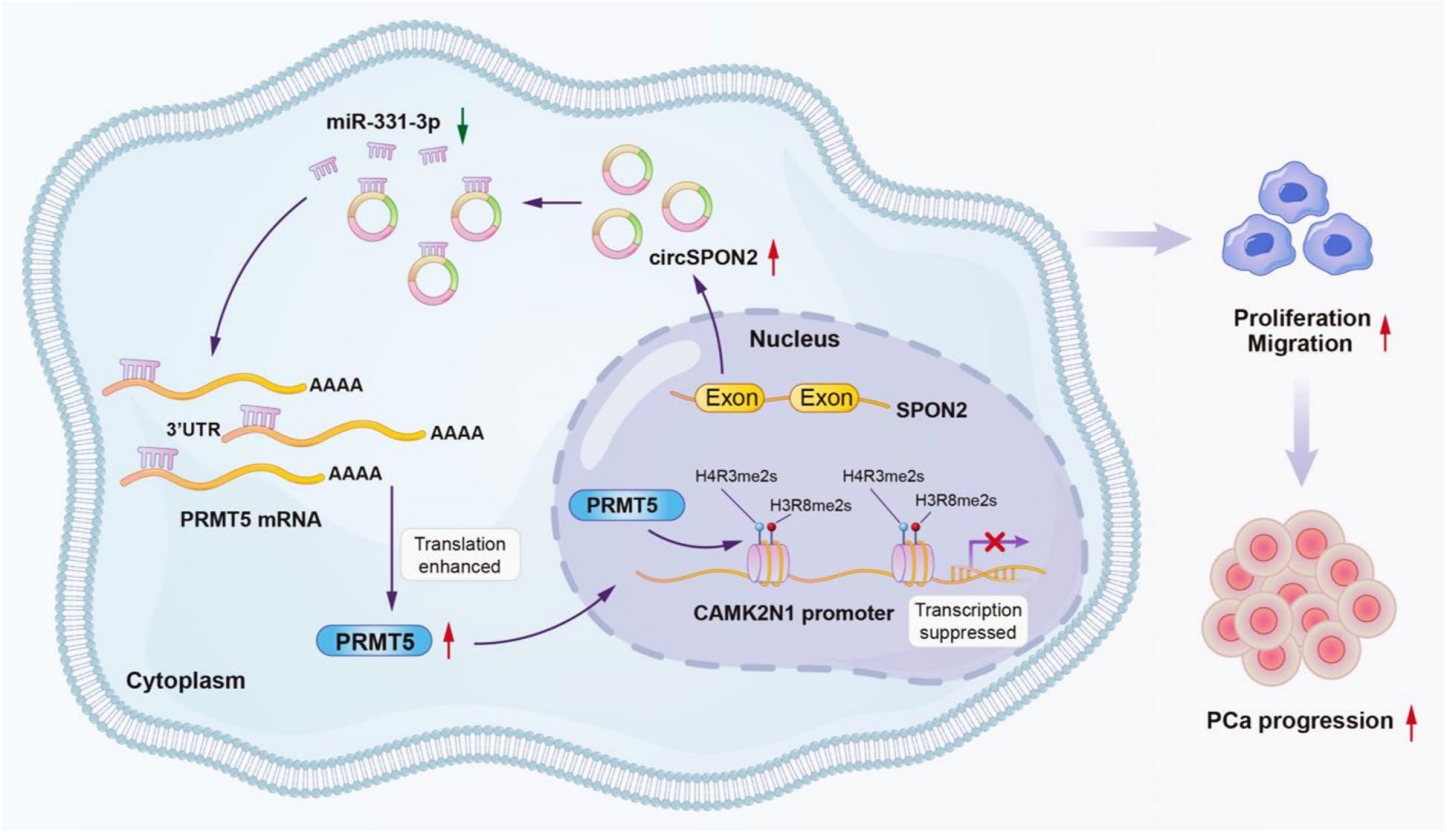
Schematic illustration indicates the mechanism by which PRMT5 aggravates PCa progression through inhibiting the transcription of CAMK2N1 and is modulated by the circSPON2/miR-331-3p axis

In summary, our findings, for the first time, revealed that the epigenetic regulator PRMT5 aggravates PCa progression through inhibiting the transcription of CAMK2N1 and is modulated by circSPON2/miR-331-3p axis (Fig. 10). This newly identified circSPON2/miR-331-3p/PRMT5/CAMK2N1 regulatory axis could be a potential therapeutic target for the treatment of aggressive PCa.

## Supplementary Information

**Additional file 1:** **Figure S1. **(A) Relative expression of CAMK2N1 levels in PCa tumor and adjacent normal tissues by qRT-PCR. (B) The correlation analysis between the expression levels of CAMK2N1 and PRMT5 in tissues from patients with PCa (n = 248; r = -0.4279, *P* < 0.01). **Figure S2. **shRNAs-mediated PRMT5 or CAMK2N1 silencing in PC-3 and DU145 cells was verified by Western blot. Hsp 70 was used as an endogenous control. **Figure S3. **(A) The mRNA levels of PRMT5 in NC or miR-331-3p-transfected PC-3 and DU145 cells were verified by qRT-PCR. Data were showed as mean ± SD. ^#^*P* > 0.05. (B) The protein levels of CAMK2N1 in NC or miR-331-3p-transfected PC-3 and DU145 cells were detected by Western blot. GAPDH was used as an endogenous control. **Figure S4. **(A)Restoration of PRMT5 expression in miR-331-3p-overexpressed PC-3 and DU145 cells was verified by qRT-PCR. (B) The expression of PRMT5 was examined by H&E staining in control, oe-miR-331-3p, oe-miR-331-3p + vector and oe-miR-331-3p + oe-PRMT5-treated xenografts tissues. Data are showed as mean ± SD. **P < 0.01. **Figure S5**. Expression profiles of circRNAs in human PCa.** (**A) Distribution of the identified circRNAs on human chromosomes. X-axis, the number of each chromosome; Y-axis, the number of circRNAs. (B) Circos plot depicting the distribution of circRNAs on human chromosomes. The outermost layer was a chromosome map of the human genome. The inner circles from outside to inside corresponded to distribution and expression of identified circRNAs on the chromosomes, distribution, and expression of significantly expressed circRNAs, respectively. (C) Composition of the identified circRNAs in terms of genomic origin. **Figure S6. **(A)The protein expression of PRMT5 was examined by H&E staining in control (NC) and circSPON2-treated xenograft tissues. Scale bar = 20 μm. (B) The mRNA expression of CAMK2N1 was determined by qRT-PCR in control (NC) and circSPON2-treated xenografts tissues. Data are showed as mean ± SD. **P < 0.01. **Figure S7.** Overexpression of circSPON2 promotes PCa cell proliferation and migration. (A) qRT-PCR analysis of circSPON2 and SPON2 mRNA in PC-3 and DU145 cells treated with vector or oe-circSPON2. (B-D) CCK-8, plate colony-formation and EdU incorporation assays were performed to determine the proliferation abilities of PC-3 and DU145 cells treated with vector or oe-circSPON2. (E) Image of subcutaneous tumor xenografts in vector or oe-circSPON2 groups. (F) The tumor growth curves of xenografts were plotted in vector or oe-circSPON2 groups. (G) The tumor weights of xenografts were evaluated. (H) Representative IHC staining micrographs of Ki-67 in tumor xenografts were conducted. Scale bar = 20 μm. (I and J) Wound healing and transwell assays were performed to determine the migratory capabilities of PC-3 and DU145 cells treated with vector or oe-circSPON2. Data were showed as mean ± SD. *P < 0.05, **P < 0.01. **Figure S8. **(A)The protein expression of PRMT5 was examined by H&E staining in control, oe-circSPON2, oe-miR-331-3p and oe-circSPON2 + oe-miR-331-3p-treated xenograft tissues. Scale bar = 20 μm. (B) The mRNA expression of CAMK2N1 was determined by qRT-PCR in control, oe-circSPON2, oe-miR-331-3p and oe-circSPON2 + oe-miR-331-3p-treated xenograft tissues. Data are showed as mean ± SD. **P < 0.01. **Table S1.** Clinicopathological characteristics of 248 PCa patients. **Table S2.** The sequences of shRNAs used in this study. **Table S3.** The sequences of primers used for real-time PCR. **Table S4.** The sequences of primers used for ChIP-qPCR. **Table S5.** Association between PRMT5 and clinicopathological features of PCa patients. **Table S6. **Association between miR-331-3p and clinicopathological features of PCa patients. **Table S7.**Differentially expressed circRNAs between five paired PCa tissues by RNA-seq. **Table S8.** Association between circSPON2 and clinicopathological features of PCa patients.

## Data Availability

The data supporting the findings of this study are available within the article and its supplementary information files and from the corresponding author upon request.

## Abbreviations

PRMT5: Protein arginine methyltransferase 5
H4R3me2s: Symmetric di-methylation at arginine 3 residue of histone H4
H3R8me2s: Symmetric di-methylation at arginine 8 residue of histone H3
CAMK2N1: Calcium/calmodulin dependent protein kinase II inhibitor 1
circRNAs: Circular RNAs
PCa: Prostate cancer
miRNAs: MicroRNAs
shRNAs: Small hairpin RNAs
FISH: Fluorescence in situ hybridization
qRT-PCR: Quantitative real-time PCR
CCK-8: Cell Counting Kit-8
EdU: 5-Ethynyl-20-deoxyuridine
ChIP: Chromatin immunoprecipitation
H&E: Hematoxylin and eosin
IHC: Immunohistochemistry
NC: Negative control
WT: Wild-type
