# Supplementary material for: The circSPON2/miR-331-3p axis regulates PRMT5, an epigenetic regulator of CAMK2N1 transcription and prostate cancer progression
Source: Mol Cancer. 2022 May 27;21:119. doi: 10.1186/s12943-022-01598-6 (PMC9137111; doi:10.1186/s12943-022-01598-6)
Supplement: Supplementary file 1 — Additional file 1: Figure S1. (A) Relative expression of CAMK2N1 levels in PCa tumor and adjacent normal tissues by qRT-PCR. (B) The correlation analysis between the expression levels of CAMK2N1 and PRMT5 in tissues from patients with PCa (n = 248; r = -0.4279, P < 0.01). Figure S2. shRNAs-mediated PRMT5 or CAMK2N1 silencing in PC-3 and DU145 cells was verified by Western blot. Hsp 70 was used as an endogenous control. Figure S3. (A) The mRNA levels of PRMT5 in NC or miR-331-3p-transfected PC-3 and DU145 cells were verified by qRT-PCR. Data were showed as mean ± SD. #P > 0.05. (B) The protein levels of CAMK2N1 in NC or miR-331-3p-transfected PC-3 and DU145 cells were detected by Western blot. GAPDH was used as an endogenous control. Figure S4. (A)Restoration of PRMT5 expression in miR-331-3p-overexpressed PC-3 and DU145 cells was verified by qRT-PCR. (B) The expression of PRMT5 was examined by H&E staining in control, oe-miR-331-3p, oe-miR-331-3p + vector and oe-miR-331-3p + oe-PRMT5-treated xenografts tissues. Data are showed as mean ± SD. **P < 0.01. Figure S5. Expression profiles of circRNAs in human PCa. (A) Distribution of the identified circRNAs on human chromosomes. X-axis, the number of each chromosome; Y-axis, the number of circRNAs. (B) Circos plot depicting the distribution of circRNAs on human chromosomes. The outermost layer was a chromosome map of the human genome. The inner circles from outside to inside corresponded to distribution and expression of identified circRNAs on the chromosomes, distribution, and expression of significantly expressed circRNAs, respectively. (C) Composition of the identified circRNAs in terms of genomic origin. Figure S6. (A)The protein expression of PRMT5 was examined by H&E staining in control (NC) and circSPON2-treated xenograft tissues. Scale bar = 20 μm. (B) The mRNA expression of CAMK2N1 was determined by qRT-PCR in control (NC) and circSPON2-treated xenografts tissues. Data are showed as mean ± SD. **P < 0.01. Figure [file 12943_2022_1598_MOESM1_ESM.docx]

**Supplemental data**


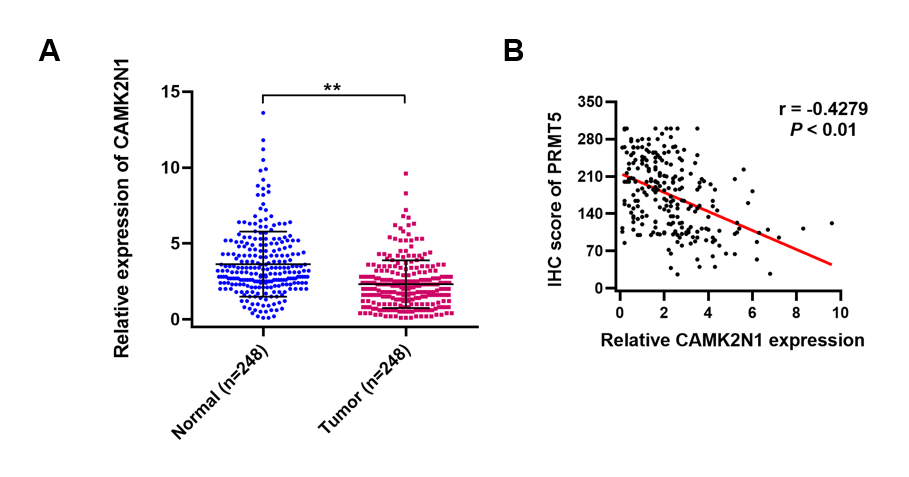


**Figure S1.** (A) Relative expression of CAMK2N1 levels in PCa tumor and adjacent normal tissues by qRT-PCR. (B) The correlation analysis between the expression levels of CAMK2N1 and PRMT5 in tissues from patients with PCa (n = 248; r = -0.4279, *P* < 0.01).


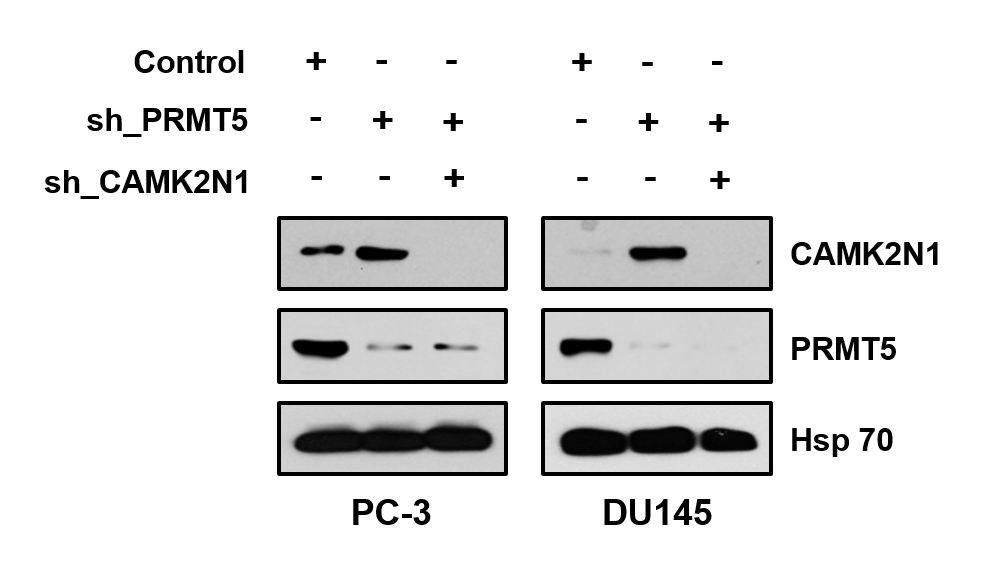


**Figure S2.** shRNAs-mediated PRMT5 or CAMK2N1 silencing in PC-3 and DU145 cells was verified by Western blot. Hsp 70 was used as an endogenous control.


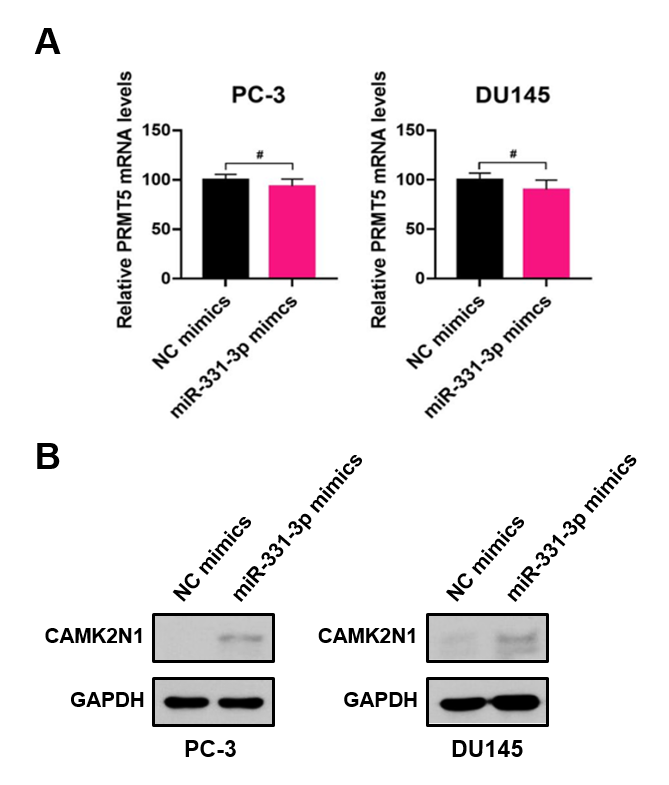


**Figure S3.** (A) The mRNA levels of PRMT5 in NC or miR-331-3p-transfected PC-3 and DU145 cells were verified by qRT-PCR. Data were showed as mean ± SD. ^#^*P* > 0.05. (B) The protein levels of CAMK2N1 in NC or miR-331-3p-transfected PC-3 and DU145 cells were detected by Western blot. GAPDH was used as an endogenous control.


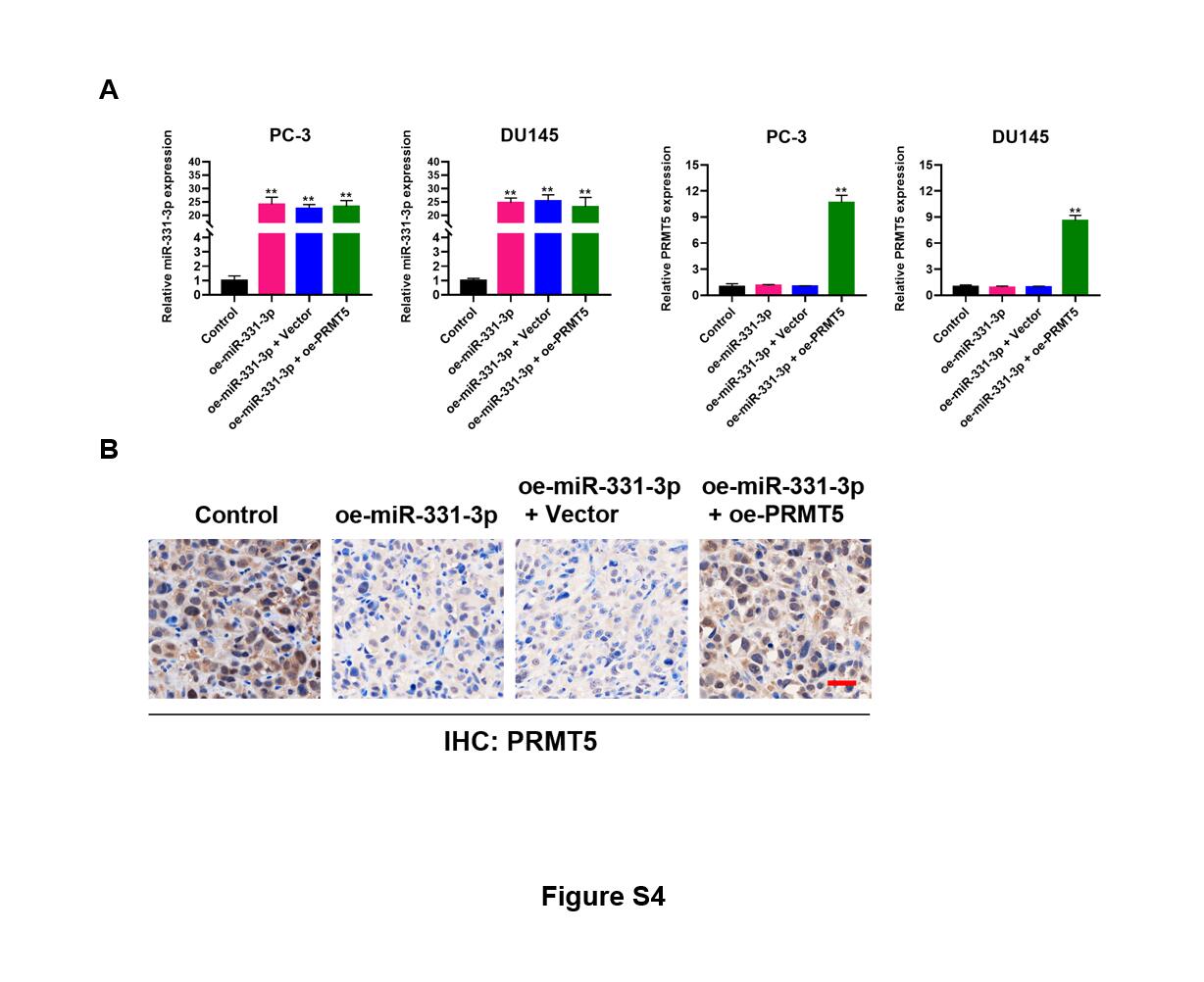


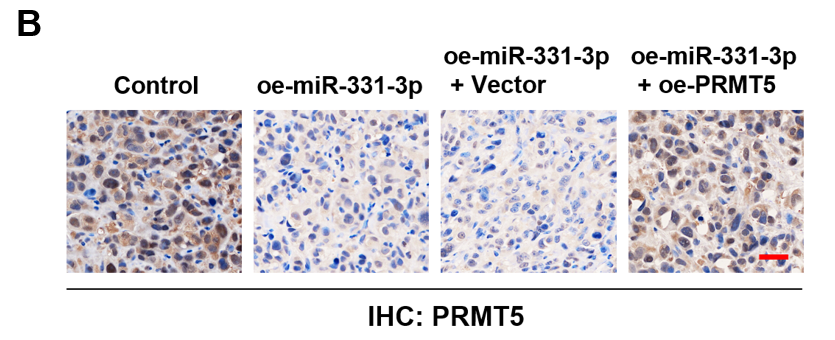


**Figure S4.** (A) Restoration of PRMT5 expression in miR-331-3p-overexpressed PC-3 and DU145 cells was verified by qRT-PCR. (B) The expression of PRMT5 was examined by H&E staining in control, oe-miR-331-3p, oe-miR-331-3p + vector and oe-miR-331-3p + oe-PRMT5-treated xenografts tissues. Data are showed as mean ± SD. ***P* < 0.01.


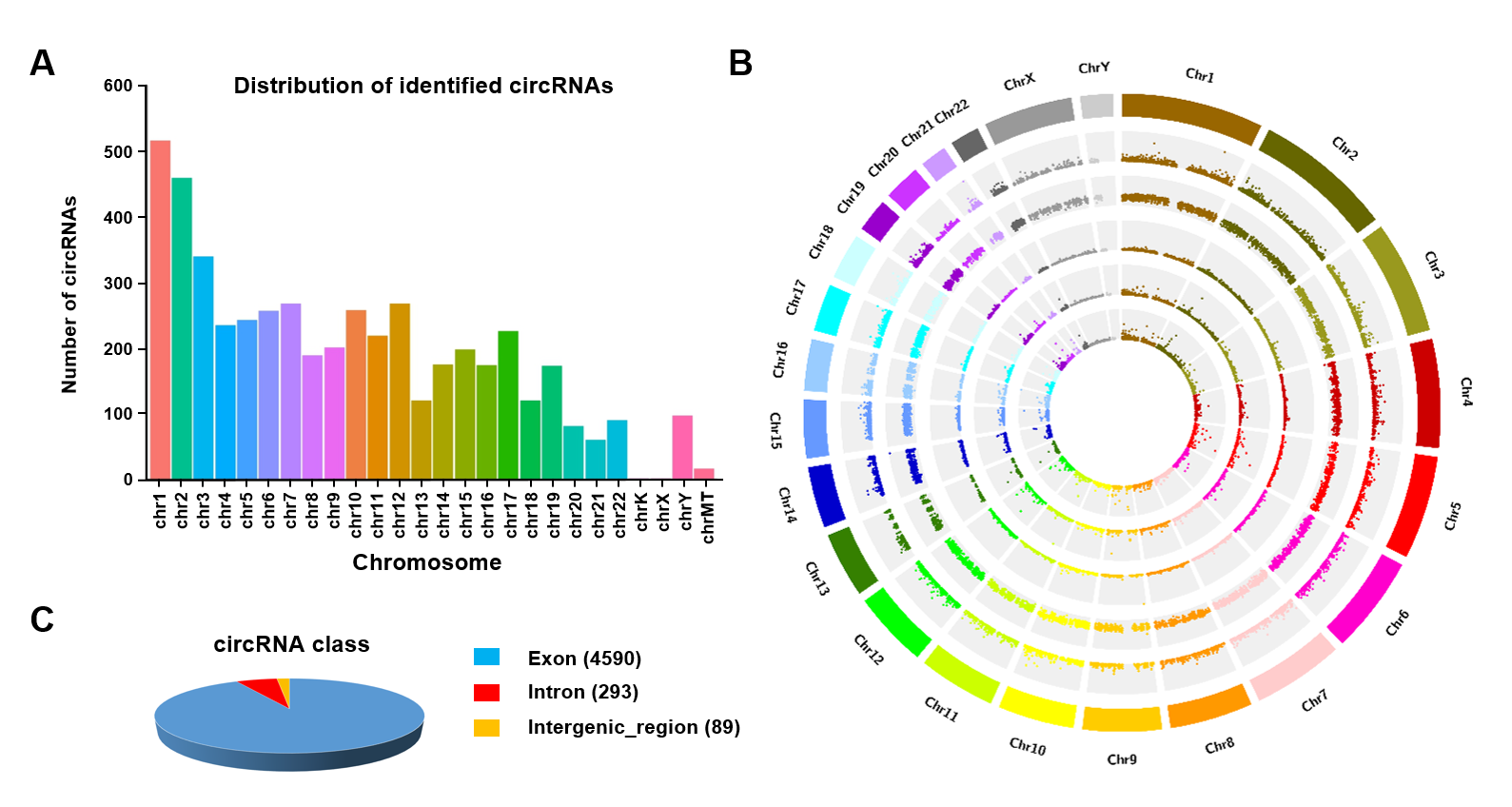


**Figure S5**. **Expression profiles of circRNAs in human PCa**. **(**A) Distribution of the identified circRNAs on human chromosomes. X-axis, the number of each chromosome; Y-axis, the number of circRNAs. (B) Circos plot depicting the distribution of circRNAs on human chromosomes. The outermost layer was a chromosome map of the human genome. The inner circles from outside to inside corresponded to distribution and expression of identified circRNAs on the chromosomes, distribution, and expression of significantly expressed circRNAs, respectively. (C) Composition of the identified circRNAs in terms of genomic origin.


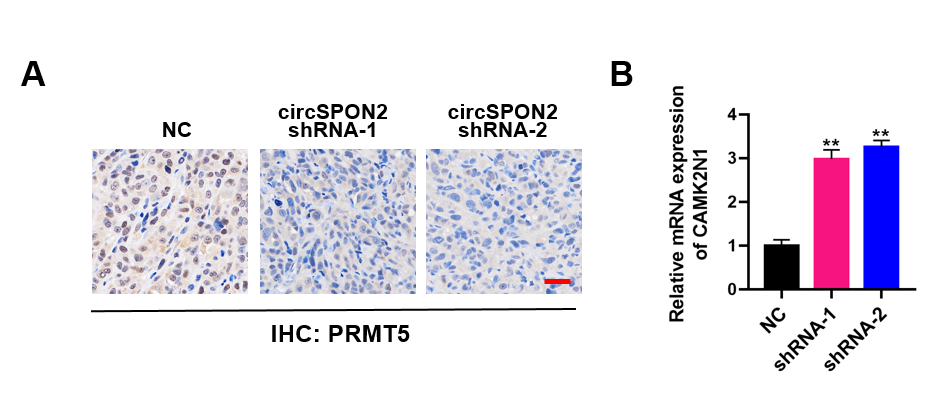


**Figure S6.** (A) The protein expression of PRMT5 was examined by H&E staining in control (NC) and circSPON2-treated xenograft tissues. Scale bar = 20 μm. (B) The mRNA expression of CAMK2N1 was determined by qRT-PCR in control (NC) and circSPON2-treated xenografts tissues. Data are showed as mean ± SD. ***P* < 0.01.


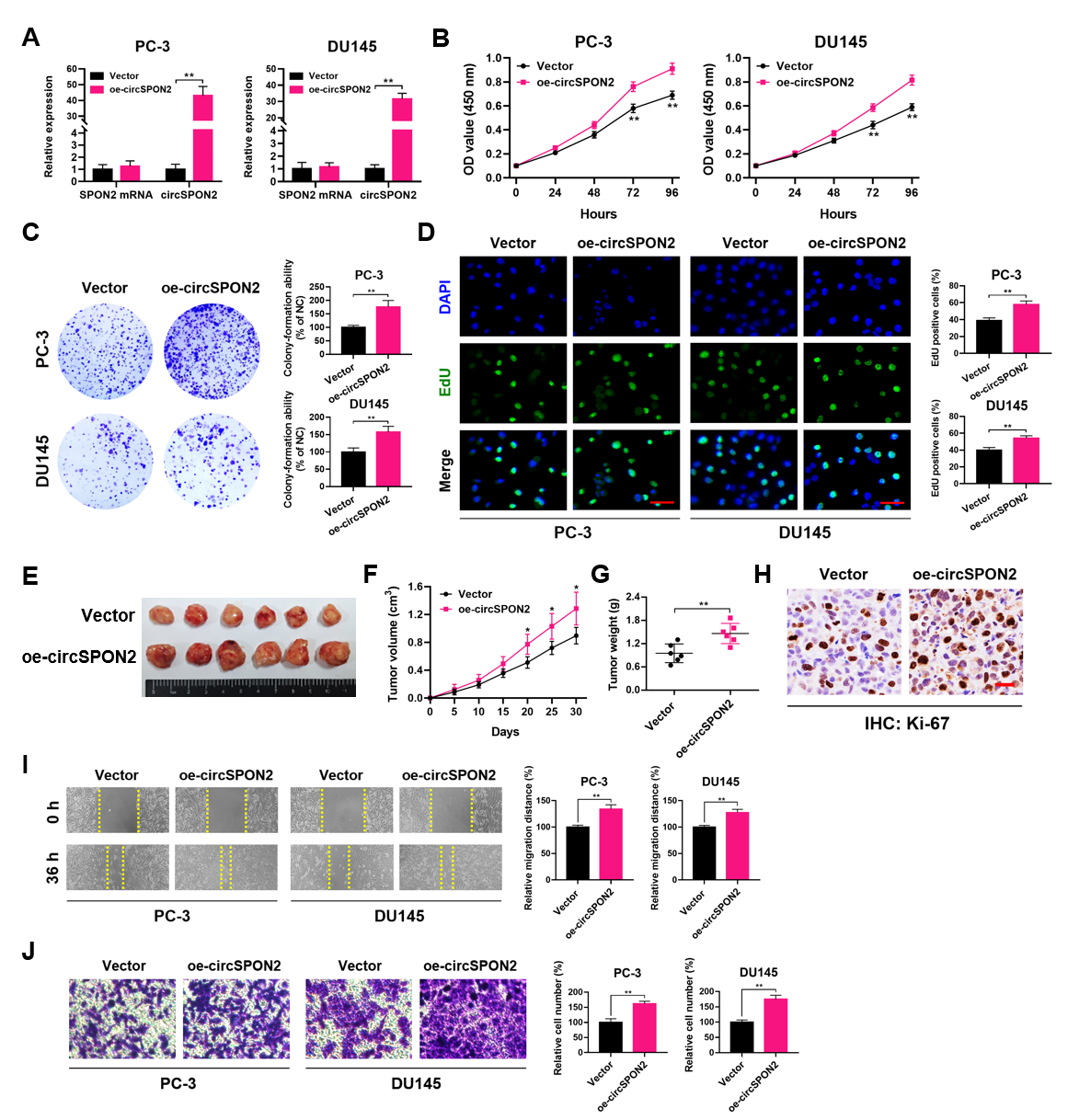


**Figure S7.** **Overexpression of circSPON2 promotes PCa cell proliferation and migration**. (A) qRT-PCR analysis of circSPON2 and SPON2 mRNA in PC-3 and DU145 cells treated with vector or oe-circSPON2. (B-D) CCK-8, plate colony-formation and EdU incorporation assays were performed to determine the proliferation abilities of PC-3 and DU145 cells treated with vector or oe-circSPON2. (E) Image of subcutaneous tumor xenografts in vector or oe-circSPON2 groups. (F) The tumor growth curves of xenografts were plotted in vector or oe-circSPON2 groups. (G) The tumor weights of xenografts were evaluated. (H) Representative IHC staining micrographs of Ki-67 in tumor xenografts were conducted. Scale bar = 20 μm. (I and J) Wound healing and transwell assays were performed to determine the migratory capabilities of PC-3 and DU145 cells treated with vector or oe-circSPON2. Data were showed as mean ± SD. **P* < 0.05, ***P* < 0.01.


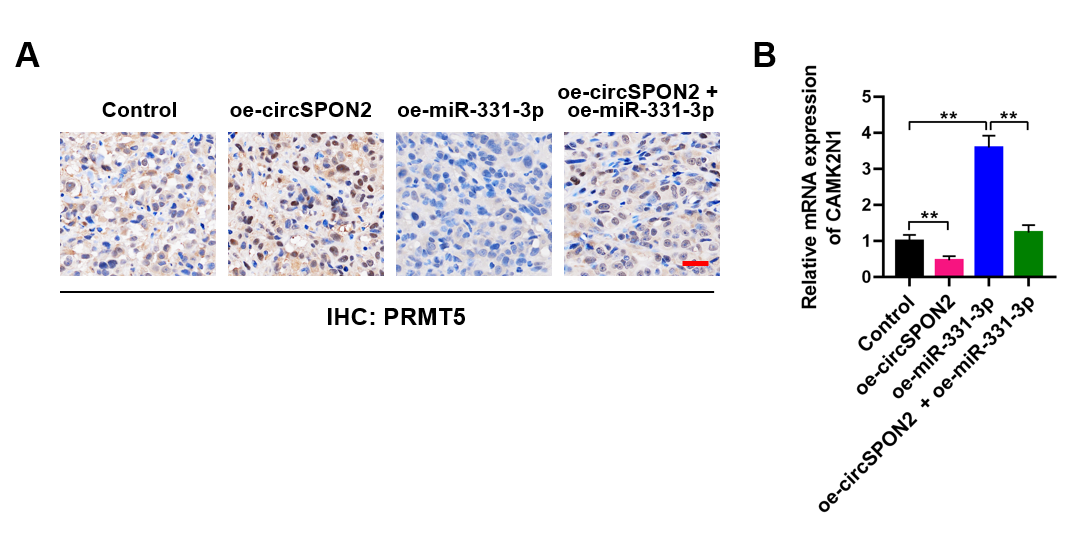


**Figure S8.** (A) The protein expression of PRMT5 was examined by H&E staining in control, oe-circSPON2, oe-miR-331-3p and oe-circSPON2 + oe-miR-331-3p-treated xenograft tissues. Scale bar = 20 μm. (B) The mRNA expression of CAMK2N1 was determined by qRT-PCR in control, oe-circSPON2, oe-miR-331-3p and oe-circSPON2 + oe-miR-331-3p-treated xenograft tissues. Data are showed as mean ± SD. ***P* < 0.01.

**Table S1. Clinicopathological characteristics of 248 PCa patients.**

| Parameters | Number of cases（%） |
| --- | --- |
| Age  <70  ≥70 | 110 (44.35%)  138 (55.65%) |
| Gleason score  5/6  7  8  9/10 | 50 (20.16%)  98 (39.52%)  52 (20.97%)  48 (19.35%) |
| T Stage  T1  T2  T3  T4 | 0 (0%)  145 (58.47%)  68 (27.42%)  35 (14.11%) |
| Lymph node metastasis  N0  N+ | 87 (35.08%)  161 (64.92%) |
| Distant metastasis  M0  M1 | 228 (91.94%)  18 (7.26%) |

**Table S2. The sequences of shRNAs used in this study.**

| shRNA | Sequence (5’-3’) |
| --- | --- |
| circSPON2 shRNA-1 | GCGGCCCTTGTGAGCAACA |
| circSPON2 shRNA-2 | TGCGCGGCCCTTGTGAGCA |
| PRMT5 shRNA-1 | GCACCAGTCTGTTCTGCTA |
| PRMT5 shRNA-2 | GGACCTGAGAGATGATATA |
| sh_CAMK2N1 | GGTTGTTATTGAAGATGAT |

**Table S3. The sequences of primers used for real-time PCR.**

| Primers | Sequence (5’-3’) |
| --- | --- |
| circSPON2 (forward) | TTTCGGAAGCGTCAGTGTTT |
| circSPON2 (reverse) | CATGCTGTAGTCGGAGCTATGC |
| PRMT5 (forward) | CTAGACCGAGTACCAGAAGAGG |
| PRMT5 (reverse) | CAGCATACAGCTTTATCCGCCG |
| CAMK2N1 (forward) | GGACACCAACAACTTCTTCGGC |
| CAMK2N1 (reverse) | GTCGGTCATATTTTTCAGCACGTC |

**Table S4. The sequences of primers used for ChIP-qPCR.**

| Primers | Sequence (5’-3’) |
| --- | --- |
| CAMK2N1 promoter P1 (forward) | CCCACCCCACCTTTTCTTCT |
| CAMK2N1 promoter P1 (reverse) | ACAGGGACTGAAAGACAGGGAG |
| CAMK2N1 promoter P2 (forward) | CCCGTAAACACCCGTGAGCG |
| CAMK2N1 promoter P2 (reverse) | GCGAGCTGCGAGGAGAAATGC |
| CAMK2N1 promoter P3 (forward) | GCCGGAGTCCAGCGCAGTT |
| CAMK2N1 promoter P3 (reverse) | GAGCCCTTCCTCCCCACCAC |
| CAMK2N1 promoter P4 (forward) | CACCTGGGCCACCTGGATGT |
| CAMK2N1 promoter P4 (reverse) | CGCTGGAGACCGTCTTTGCT |
| CAMK2N1 promoter P5 (forward) | AGTGCTACCGTTCTTTCTCA |
| CAMK2N1 promoter P5 (reverse) | CCCGTGGTTTGCTGACTG |
| CAMK2N1 promoter P6 (forward) | TTATCATCCTGCTTGTTACGC |
| CAMK2N1 promoter P6 (reverse) | CTGGCTCATTCATTAGGACTTT |
| CAMK2N1 promoter P7 (forward) | GGAACAAGGCAAGGTAAACA |
| CAMK2N1 promoter P7 (reverse) | GAAAGACCCAGCGAGGAC |

**Table S5. Association between PRMT5 and clinicopathological features of PCa patients.**

| Characteristics | Cases (%) | PRMT5 expression | *P* value |
| --- | --- | --- | --- |
| Age |  |  |  |
| <70 | 110 (44.35%) | 175.36 ± 70.78 | 0.8255 |
| ≥70 | 138 (55.65%) | 173.51 ± 61.52 |  |
| Gleason score |  |  |  |
| 5-7 | 148 (59.68%) | 151.86 ± 58.00 | **0.000*** |
| 8-10 | 100 (40.32%) | 207.58 ± 62.37 |  |
| T stage |  |  |  |
| T1-2 | 145 (58.47%) | 139.19 ± 53.20 | **0.000*** |
| T3-4 | 103 (41.32%) | 224.72 ± 47.09 |  |
| Lymph node metastasis |  |  |  |
| N0 | 87 (35.08%) | 173.40 ± 63.26 | 0.8704 |
| N+ | 161 (64.92%) | 174.83 ± 67.11 |  |
| Distant metastasis |  |  |  |
| M0 | 228 (91.94%) | 173.72 ± 65.46 | 0.6914 |
| M1 | 18 (7.26%) | 180.11 ± 68.78 |  |

**P* < 0.05 is considered significant.

**Table S6. Association between miR-331-3p and clinicopathological features of PCa patients.**

| Characteristics | Cases (%) | miR-331-3p expression | *P* value |
| --- | --- | --- | --- |
| Age |  |  |  |
| <70 | 110 (44.35%) | 3.77 ± 3.25 | 0.8343 |
| ≥70 | 138 (55.65%) | 3.68 ± 3.27 |  |
| Gleason score |  |  |  |
| 5-7 | 148 (59.68%) | 5.23 ± 3.29 | **0.000*** |
| 8-10 | 100 (40.32%) | 1.50 ± 1.39 |  |
| T stage |  |  |  |
| T1-2 | 145 (58.47%) | 4.63 ± 3.30 | **0.000*** |
| T3-4 | 103 (41.32%) | 2.45 ± 2.74 |  |
| Lymph node metastasis |  |  |  |
| N0 | 87 (35.08%) | 3.67 ± 3.20 | 0.8594 |
| N+ | 161 (64.92%) | 3.75 ± 3.29 |  |
| Distant metastasis |  |  |  |
| M0 | 228 (91.94%) | 3.75 ± 3.16 | 0.9839 |
| M1 | 18 (7.26%) | 3.73 ± 4.41 |  |

**P* < 0.05 is considered significant.

**Table S7. Differentially expressed circRNAs between five paired PCa tissues by RNA-seq.**

| CIRI_ID | log2(Fold Change  (PC_T/PC_N)) | P value | Regulate | Gene_ID | Gene_name |
| --- | --- | --- | --- | --- | --- |
| CIRI_circ_10915 | 8.374155968 | 0.005707 | up | ENSG00000159674 | SPON2 |
| CIRI_circ_13495 | 7.400645381 | 0.011066 | up | ENSG00000166743 | ACSM1 |
| CIRI_circ_3052 | 6.234029416 | 0.001313 | up | _ | _ |
| CIRI_circ_4991 | 6.135295273 | 0.043708 | up | ENSG00000104413 | ESRP1 |
| CIRI_circ_2528 | 6.033600906 | 0.0474 | up | _ | _ |
| CIRI_circ_11319 | 6.032498695 | 0.025151 | up | ENSG00000138696 | BMPR1B |
| CIRI_circ_7916 | 5.975397051 | 0.020315 | up | ENSG00000285837 | AC024598.1 |
| CIRI_circ_1603 | 5.963181593 | 0.02363 | up | ENSG00000215018 | COL28A1 |
| CIRI_circ_13932 | 5.958873385 | 0.000108 | up | ENSG00000139865 | TTC6 |
| CIRI_circ_15267 | 5.517060347 | 0.001908 | up | ENSG00000177694 | NAALADL2 |
| CIRI_circ_7037 | 5.512599931 | 0.002107 | up | ENSG00000169410 | PTPN9 |
| CIRI_circ_11801 | 5.45306033 | 0.029659 | up | ENSG00000242110 | AMACR |
| CIRI_circ_5536 | 5.400462839 | 0.002825 | up | ENSG00000163694 | RBM47 |
| CIRI_circ_5210 | 5.274651531 | 0.005129 | up | _ | _ |
| CIRI_circ_13584 | 5.267603629 | 0.000815 | up | ENSG00000080493 | SLC4A4 |
| CIRI_circ_13368 | 5.252360055 | 0.01987 | up | ENSG00000149639 | SOGA1 |
| CIRI_circ_13390 | 5.252360055 | 0.01987 | up | ENSG00000172915 | NBEA |
| CIRI_circ_14494 | 5.252360055 | 0.01987 | up | ENSG00000152818 | UTRN |
| CIRI_circ_5257 | 5.252360055 | 0.01987 | up | ENSG00000109586 | GALNT7 |
| CIRI_circ_7417 | 5.236367148 | 0.005336 | up | ENSG00000183077 | AFMID |
| CIRI_circ_14160 | 5.232460939 | 0.011056 | up | _ | _ |
| CIRI_circ_5578 | 5.215973818 | 0.02889 | up | _ | _ |
| CIRI_circ_14072 | 5.197395701 | 0.012096 | up | ENSG00000127663 | KDM4B |
| CIRI_circ_11639 | 5.163546984 | 0.024401 | up | ENSG00000105186 | ANKRD27 |
| CIRI_circ_5322 | 5.131705283 | 8.34E-05 | up | ENSG00000169710 | FASN |
| CIRI_circ_9893 | 5.11751672 | 0.015295 | up | ENSG00000038382 | TRIO |
| CIRI_circ_13053 | 5.113577997 | 0.00767 | up | _ | _ |
| CIRI_circ_12648 | 5.05597995 | 0.029825 | up | ENSG00000196353 | CPNE4 |
| CIRI_circ_13362 | 5.05597995 | 0.029825 | up | ENSG00000154914 | USP43 |
| CIRI_circ_8733 | 5.05597995 | 0.029825 | up | ENSG00000004534 | RBM6 |
| CIRI_circ_13363 | 5.052875322 | 0.018068 | up | ENSG00000136205 | TNS3 |
| CIRI_circ_11194 | 5.048807299 | 0.017282 | up | ENSG00000172765 | TMCC1 |
| CIRI_circ_3770 | 5.01468539 | 0.033534 | up | _ | _ |
| CIRI_circ_7823 | 4.998319394 | 0.019822 | up | ENSG00000021776 | AQR |
| CIRI_circ_2139 | 4.992020465 | 0.034517 | up | ENSG00000103534 | TMC5 |
| CIRI_circ_3256 | 4.992020465 | 0.034517 | up | ENSG00000105983 | LMBR1 |
| CIRI_circ_16105 | 4.959916685 | 0.011098 | up | ENSG00000188372 | ZP3 |
| CIRI_circ_6476 | 4.924136501 | 0.023359 | up | ENSG00000138696 | BMPR1B |
| CIRI_circ_12502 | 4.91791556 | 0.023635 | up | ENSG00000172954 | LCLAT1 |
| CIRI_circ_10302 | 4.911322112 | 0.041348 | up | ENSG00000242715 | CCDC169 |
| CIRI_circ_605 | 4.880294884 | 0.025924 | up | ENSG00000196730 | DAPK1 |
| CIRI_circ_11936 | 4.865122464 | 0.027186 | up | ENSG00000102900 | NUP93 |
| CIRI_circ_11846 | 4.862566552 | 0.044925 | up | ENSG00000251192 | ZNF674 |
| CIRI_circ_9090 | 4.862566552 | 0.044925 | up | ENSG00000137802 | MAPKBP1 |
| CIRI_circ_7244 | 4.84689774 | 0.026184 | up | ENSG00000166169 | POLL |
| CIRI_circ_585 | 4.82956363 | 0.028571 | up | ENSG00000142515 | KLK3 |
| CIRI_circ_1864 | 4.829367885 | 0.045982 | up | ENSG00000287460 | AL442636.1 |
| CIRI_circ_1965 | 4.829367885 | 0.045982 | up | _ | _ |
| CIRI_circ_4560 | 4.829367885 | 0.045982 | up | ENSG00000185621 | LMLN |
| CIRI_circ_5228 | 4.829367885 | 0.045982 | up | ENSG00000021574 | SPAST |
| CIRI_circ_6880 | 4.829367885 | 0.045982 | up | ENSG00000080493 | SLC4A4 |
| CIRI_circ_8280 | 4.829367885 | 0.045982 | up | ENSG00000144445 | KANSL1L |
| CIRI_circ_9847 | 4.829367885 | 0.045982 | up | ENSG00000137075 | RNF38 |
| CIRI_circ_14696 | 4.820875055 | 0.016044 | up | ENSG00000067208 | EVI5 |
| CIRI_circ_7796 | 4.806401304 | 0.029782 | up | ENSG00000224078 | SNHG14 |
| CIRI_circ_10381 | 4.797601361 | 0.049745 | up | ENSG00000215472 | RPL17-C18orf32 |
| CIRI_circ_11725 | 4.797601361 | 0.049745 | up | ENSG00000009694 | TENM1 |
| CIRI_circ_15711 | 4.797601361 | 0.049745 | up | ENSG00000197818 | SLC9A8 |
| CIRI_circ_1582 | 4.797601361 | 0.049745 | up | ENSG00000163539 | CLASP2 |
| CIRI_circ_3646 | 4.797601361 | 0.049745 | up | _ | _ |
| CIRI_circ_4171 | 4.797601361 | 0.049745 | up | ENSG00000119772 | DNMT3A |
| CIRI_circ_11716 | 4.777337238 | 0.030931 | up | ENSG00000033011 | ALG1 |
| CIRI_circ_12716 | 4.762036766 | 0.033425 | up | ENSG00000096060 | FKBP5 |
| CIRI_circ_13745 | 4.760730702 | 0.019413 | up | ENSG00000165097 | KDM1B |
| CIRI_circ_186 | 4.701930349 | 0.001942 | up | ENSG00000288591 | AC078815.1 |
| CIRI_circ_10837 | 4.680470446 | 0.004559 | up | ENSG00000139865 | TTC6 |
| CIRI_circ_5226 | 4.672898104 | 0.040684 | up | ENSG00000188959 | C9orf152 |
| CIRI_circ_9836 | 4.672898104 | 0.040684 | up | ENSG00000103126 | AXIN1 |
| CIRI_circ_10495 | 4.65524319 | 0.040031 | up | ENSG00000165102 | HGSNAT |
| CIRI_circ_13525 | 4.636529747 | 0.044578 | up | ENSG00000105738 | SIPA1L3 |
| CIRI_circ_12891 | 4.628121778 | 0.001215 | up | ENSG00000049759 | NEDD4L |
| CIRI_circ_11377 | 4.608245828 | 0.046193 | up | ENSG00000145349 | CAMK2D |
| CIRI_circ_456 | 4.598759855 | 0.045484 | up | ENSG00000085719 | CPNE3 |
| CIRI_circ_5547 | 4.595753181 | 0.046524 | up | ENSG00000140527 | WDR93 |
| CIRI_circ_1617 | 4.193963926 | 0.018173 | up | _ | _ |
| CIRI_circ_8277 | 4.14821985 | 0.009858 | up | ENSG00000127663 | KDM4B |
| CIRI_circ_9021 | 4.126032751 | 0.008613 | up | ENSG00000138346 | DNA2 |
| CIRI_circ_8207 | 4.112657483 | 0.03944 | up | _ | _ |
| CIRI_circ_5849 | 4.106321125 | 0.02177 | up | ENSG00000059758 | CDK17 |
| CIRI_circ_7854 | 3.936007078 | 0.003617 | up | ENSG00000271254 | AC240274.1 |
| CIRI_circ_3625 | 3.852053896 | 0.004009 | up | ENSG00000172081 | MOB3A |
| CIRI_circ_3572 | 3.798647852 | 0.037967 | up | ENSG00000168137 | SETD5 |
| CIRI_circ_15007 | 3.666396834 | 0.007163 | up | ENSG00000106617 | PRKAG2 |
| CIRI_circ_367 | 3.654586144 | 0.013498 | up | ENSG00000065485 | PDIA5 |
| CIRI_circ_11029 | 3.634789667 | 0.003711 | up | ENSG00000165325 | DEUP1 |
| CIRI_circ_8476 | 3.614803845 | 0.03764 | up | ENSG00000278540 | ACACA |
| CIRI_circ_15896 | 3.51848132 | 2.59E-08 | up | ENSG00000182795 | C1orf116 |
| CIRI_circ_4314 | 3.428585511 | 0.012328 | up | ENSG00000145734 | BDP1 |
| CIRI_circ_10551 | 3.218262006 | 0.003453 | up | ENSG00000204385 | SLC44A4 |
| CIRI_circ_3687 | 3.156916584 | 1.78E-08 | up | ENSG00000184012 | TMPRSS2 |
| CIRI_circ_9879 | 3.143073414 | 0.011649 | up | ENSG00000179832 | MROH1 |
| CIRI_circ_6583 | 3.104102917 | 0.039258 | up | ENSG00000127663 | KDM4B |
| CIRI_circ_11540 | 3.10122101 | 0.008607 | up | ENSG00000184012 | TMPRSS2 |
| CIRI_circ_11350 | 2.948686869 | 0.016425 | up | ENSG00000140526 | ABHD2 |
| CIRI_circ_5896 | 2.890242552 | 0.000768 | up | ENSG00000168743 | NPNT |
| CIRI_circ_4509 | 2.886186535 | 0.007772 | up | ENSG00000080493 | SLC4A4 |
| CIRI_circ_2978 | 2.86840825 | 0.000111 | up | ENSG00000143797 | MBOAT2 |
| CIRI_circ_8363 | 2.800061188 | 0.007059 | up | ENSG00000130529 | TRPM4 |
| CIRI_circ_7293 | 2.641326105 | 0.003584 | up | _ | _ |
| CIRI_circ_9277 | 2.612691229 | 0.001941 | up | ENSG00000146555 | SDK1 |
| CIRI_circ_10681 | 2.584717146 | 0.001476 | up | ENSG00000137571 | SLCO5A1 |
| CIRI_circ_7700 | 2.519653352 | 0.00802 | up | ENSG00000086991 | NOX4 |
| CIRI_circ_244 | 2.511126096 | 0.032193 | up | ENSG00000009694 | TENM1 |
| CIRI_circ_7176 | 2.472397788 | 0.025857 | up | ENSG00000237438 | CECR7 |
| CIRI_circ_12406 | 2.456874192 | 0.007556 | up | ENSG00000110931 | CAMKK2 |
| CIRI_circ_4332 | 2.385090439 | 0.006212 | up | ENSG00000138696 | BMPR1B |
| CIRI_circ_13985 | 2.360869503 | 0.002241 | up | ENSG00000143797 | MBOAT2 |
| CIRI_circ_6798 | 2.351263506 | 0.0008 | up | ENSG00000162105 | SHANK2 |
| CIRI_circ_11883 | 2.314285463 | 3.79E-05 | up | ENSG00000049759 | NEDD4L |
| CIRI_circ_15486 | 2.310513379 | 0.003118 | up | ENSG00000083223 | TUT7 |
| CIRI_circ_13125 | 2.241908239 | 0.008945 | up | ENSG00000146067 | FAM193B |
| CIRI_circ_1764 | 2.22370136 | 0.001024 | up | ENSG00000105514 | RAB3D |
| CIRI_circ_919 | 2.213096139 | 0.045067 | up | ENSG00000108239 | TBC1D12 |
| CIRI_circ_4227 | 2.211677575 | 0.006885 | up | ENSG00000173715 | C11orf80 |
| CIRI_circ_7869 | 2.165035689 | 0.020881 | up | _ | _ |
| CIRI_circ_13705 | 2.153520536 | 0.000599 | up | ENSG00000143797 | MBOAT2 |
| CIRI_circ_9915 | 2.118619795 | 0.001214 | up | ENSG00000143797 | MBOAT2 |
| CIRI_circ_8349 | 2.092233268 | 0.023714 | up | ENSG00000181789 | COPG1 |
| CIRI_circ_15001 | 2.064873265 | 0.038481 | up | ENSG00000188554 | NBR1 |
| CIRI_circ_4104 | 1.978689745 | 0.037404 | up | ENSG00000185359 | HGS |
| CIRI_circ_1880 | 1.978387715 | 0.026648 | up | ENSG00000124406 | ATP8A1 |
| CIRI_circ_4291 | 1.926204586 | 0.00792 | up | ENSG00000144036 | EXOC6B |
| CIRI_circ_11614 | 1.913458621 | 0.024483 | up | ENSG00000131844 | MCCC2 |
| CIRI_circ_9676 | 1.8753438 | 0.020822 | up | ENSG00000017260 | ATP2C1 |
| CIRI_circ_1007 | 1.85857129 | 0.019335 | up | ENSG00000155660 | PDIA4 |
| CIRI_circ_9239 | 1.777544754 | 0.021361 | up | ENSG00000152217 | SETBP1 |
| CIRI_circ_13122 | 1.775581885 | 0.014603 | up | ENSG00000008382 | MPND |
| CIRI_circ_1291 | 1.669337392 | 0.013006 | up | ENSG00000137073 | UBAP2 |
| CIRI_circ_9723 | 1.643574219 | 0.004345 | up | ENSG00000100116 | GCAT |
| CIRI_circ_10228 | 1.575945986 | 0.000713 | up | ENSG00000140526 | ABHD2 |
| CIRI_circ_7526 | 1.540307349 | 0.001816 | up | ENSG00000113384 | GOLPH3 |
| CIRI_circ_7160 | 1.525597752 | 0.033196 | up | ENSG00000132874 | SLC14A2 |
| CIRI_circ_2063 | 1.438698864 | 0.030874 | up | ENSG00000113719 | ERGIC1 |
| CIRI_circ_8715 | 1.417911396 | 0.019399 | up | ENSG00000185624 | P4HB |
| CIRI_circ_11086 | 1.37437982 | 0.010355 | up | ENSG00000127663 | KDM4B |
| CIRI_circ_15193 | 1.332391198 | 0.019751 | up | ENSG00000263001 | GTF2I |
| CIRI_circ_12903 | 1.259552155 | 0.00408 | up | ENSG00000142197 | DOP1B |
| CIRI_circ_11059 | 1.212800818 | 0.013341 | up | ENSG00000083223 | TUT7 |
| CIRI_circ_13221 | 1.184763614 | 0.028936 | up | ENSG00000152818 | UTRN |
| CIRI_circ_11839 | 1.078350543 | 0.024701 | up | ENSG00000131149 | GSE1 |
| CIRI_circ_2587 | -1.121536519 | 0.022324 | down | ENSG00000140718 | FTO |
| CIRI_circ_642 | -1.143594535 | 0.044437 | down | ENSG00000109381 | ELF2 |
| CIRI_circ_14023 | -1.177188064 | 0.004421 | down | ENSG00000117868 | ESYT2 |
| CIRI_circ_16042 | -1.194226359 | 0.017677 | down | ENSG00000110422 | HIPK3 |
| CIRI_circ_2900 | -1.216736743 | 0.013768 | down | ENSG00000198121 | LPAR1 |
| CIRI_circ_13453 | -1.252604416 | 0.011376 | down | ENSG00000134744 | TUT4 |
| CIRI_circ_280 | -1.271455965 | 0.034726 | down | ENSG00000165322 | ARHGAP12 |
| CIRI_circ_2331 | -1.308837868 | 0.034827 | down | ENSG00000141627 | DYM |
| CIRI_circ_408 | -1.331034583 | 0.040549 | down | ENSG00000057608 | GDI2 |
| CIRI_circ_5603 | -1.339942323 | 0.038878 | down | ENSG00000113658 | SMAD5 |
| CIRI_circ_15223 | -1.366277881 | 0.020415 | down | ENSG00000137478 | FCHSD2 |
| CIRI_circ_16295 | -1.370353641 | 0.033029 | down | ENSG00000157110 | RBPMS |
| CIRI_circ_1293 | -1.414926945 | 0.049569 | down | ENSG00000136104 | RNASEH2B |
| CIRI_circ_5233 | -1.428727007 | 0.011783 | down | ENSG00000169193 | CCDC126 |
| CIRI_circ_6687 | -1.458787139 | 0.024694 | down | ENSG00000102893 | PHKB |
| CIRI_circ_12979 | -1.462069124 | 0.028644 | down | ENSG00000169398 | PTK2 |
| CIRI_circ_14964 | -1.473377667 | 0.001857 | down | ENSG00000276087 | AC008073.3 |
| CIRI_circ_6106 | -1.509983423 | 0.016421 | down | ENSG00000112992 | NNT |
| CIRI_circ_52 | -1.544543377 | 0.046451 | down | ENSG00000101019 | UQCC1 |
| CIRI_circ_7639 | -1.559971461 | 0.026581 | down | ENSG00000146247 | PHIP |
| CIRI_circ_3422 | -1.56066024 | 0.03189 | down | ENSG00000122507 | BBS9 |
| CIRI_circ_10197 | -1.561447031 | 0.038814 | down | ENSG00000132383 | RPA1 |
| CIRI_circ_8307 | -1.616101905 | 0.031511 | down | ENSG00000071205 | ARHGAP10 |
| CIRI_circ_5503 | -1.638780795 | 0.041421 | down | ENSG00000116489 | CAPZA1 |
| CIRI_circ_10590 | -1.656372241 | 0.013469 | down | ENSG00000112320 | SOBP |
| CIRI_circ_5952 | -1.65756809 | 0.007085 | down | ENSG00000197442 | MAP3K5 |
| CIRI_circ_14990 | -1.658810759 | 0.029578 | down | ENSG00000103351 | CLUAP1 |
| CIRI_circ_8489 | -1.660070796 | 0.002293 | down | ENSG00000103591 | AAGAB |
| CIRI_circ_14526 | -1.679013696 | 0.03119 | down | ENSG00000022567 | SLC45A4 |
| CIRI_circ_16039 | -1.698306824 | 0.037929 | down | ENSG00000172292 | CERS6 |
| CIRI_circ_11536 | -1.711136425 | 0.023153 | down | ENSG00000147854 | UHRF2 |
| CIRI_circ_530 | -1.713831449 | 0.024522 | down | ENSG00000136169 | SETDB2 |
| CIRI_circ_2869 | -1.753400979 | 0.021653 | down | ENSG00000075945 | KIFAP3 |
| CIRI_circ_3950 | -1.757909372 | 0.049997 | down | ENSG00000240038 | AMY2B |
| CIRI_circ_15154 | -1.784428428 | 0.009978 | down | ENSG00000196418 | ZNF124 |
| CIRI_circ_16083 | -1.794929209 | 0.045858 | down | ENSG00000122557 | HERPUD2 |
| CIRI_circ_10748 | -1.798300734 | 0.047455 | down | ENSG00000178971 | CTC1 |
| CIRI_circ_8025 | -1.823666559 | 0.018666 | down | ENSG00000151322 | NPAS3 |
| CIRI_circ_6173 | -1.830994927 | 0.007712 | down | ENSG00000130638 | ATXN10 |
| CIRI_circ_7870 | -1.831062273 | 0.021798 | down | ENSG00000169398 | PTK2 |
| CIRI_circ_13722 | -1.856481495 | 0.036328 | down | ENSG00000197386 | HTT |
| CIRI_circ_4413 | -1.867485475 | 0.024335 | down | ENSG00000084676 | NCOA1 |
| CIRI_circ_6317 | -1.871952215 | 0.019702 | down | ENSG00000131873 | CHSY1 |
| CIRI_circ_10927 | -1.879809451 | 0.019683 | down | ENSG00000173258 | ZNF483 |
| CIRI_circ_15175 | -1.948262126 | 0.03023 | down | ENSG00000126216 | TUBGCP3 |
| CIRI_circ_13980 | -1.95433499 | 0.020677 | down | ENSG00000248643 | RBM14-RBM4 |
| CIRI_circ_14589 | -1.976374206 | 0.046577 | down | ENSG00000103319 | EEF2K |
| CIRI_circ_4855 | -1.979397432 | 0.007193 | down | ENSG00000160321 | ZNF208 |
| CIRI_circ_4034 | -1.989104511 | 0.005219 | down | ENSG00000196323 | ZBTB44 |
| CIRI_circ_10193 | -1.995193778 | 0.045691 | down | ENSG00000114107 | CEP70 |
| CIRI_circ_12058 | -2.04583883 | 0.016524 | down | ENSG00000113319 | RASGRF2 |
| CIRI_circ_5570 | -2.056872588 | 0.047291 | down | ENSG00000110090 | CPT1A |
| CIRI_circ_12705 | -2.05881088 | 0.017536 | down | ENSG00000135749 | PCNX2 |
| CIRI_circ_8245 | -2.106676637 | 0.010808 | down | _ | _ |
| CIRI_circ_12190 | -2.163229993 | 0.026731 | down | ENSG00000100722 | ZC3H14 |
| CIRI_circ_3022 | -2.216838764 | 0.003214 | down | ENSG00000005889 | ZFX |
| CIRI_circ_12625 | -2.218487871 | 0.007971 | down | ENSG00000131508 | UBE2D2 |
| CIRI_circ_4676 | -2.248953081 | 0.033354 | down | ENSG00000138134 | STAMBPL1 |
| CIRI_circ_13802 | -2.24984618 | 0.048604 | down | ENSG00000145687 | SSBP2 |
| CIRI_circ_10582 | -2.256947325 | 0.017104 | down | ENSG00000120071 | KANSL1 |
| CIRI_circ_1367 | -2.340887284 | 0.041313 | down | ENSG00000066382 | MPPED2 |
| CIRI_circ_15698 | -2.355092929 | 0.001479 | down | ENSG00000107518 | ATRNL1 |
| CIRI_circ_8165 | -2.397415352 | 0.049161 | down | ENSG00000065154 | OAT |
| CIRI_circ_8893 | -2.400096934 | 0.016609 | down | ENSG00000137776 | SLTM |
| CIRI_circ_12361 | -2.415718866 | 0.044192 | down | ENSG00000137513 | NARS2 |
| CIRI_circ_1934 | -2.430954641 | 0.035975 | down | ENSG00000166889 | PATL1 |
| CIRI_circ_9483 | -2.476269912 | 0.035404 | down | ENSG00000146909 | NOM1 |
| CIRI_circ_12595 | -2.479960016 | 0.005924 | down | ENSG00000100154 | TTC28 |
| CIRI_circ_6794 | -2.503970459 | 0.002687 | down | ENSG00000112851 | ERBIN |
| CIRI_circ_1572 | -2.540915037 | 0.043161 | down | ENSG00000146151 | HMGCLL1 |
| CIRI_circ_3877 | -2.542796902 | 0.000207 | down | ENSG00000135424 | ITGA7 |
| CIRI_circ_3551 | -2.574342255 | 0.026583 | down | ENSG00000272980 | Z94721.2 |
| CIRI_circ_5515 | -2.606095438 | 0.034636 | down | ENSG00000186001 | LRCH3 |
| CIRI_circ_16377 | -2.614164104 | 0.046542 | down | ENSG00000237877 | LINC01473 |
| CIRI_circ_16120 | -2.667417055 | 0.009607 | down | ENSG00000132964 | CDK8 |
| CIRI_circ_15353 | -2.67925478 | 0.012052 | down | ENSG00000149289 | ZC3H12C |
| CIRI_circ_6572 | -2.715288768 | 0.048504 | down | ENSG00000144559 | TAMM41 |
| CIRI_circ_1653 | -2.72866052 | 0.034023 | down | ENSG00000185483 | ROR1 |
| CIRI_circ_1140 | -2.737064969 | 0.044781 | down | ENSG00000213047 | DENND1B |
| CIRI_circ_5384 | -2.767472453 | 0.030097 | down | ENSG00000198780 | FAM169A |
| CIRI_circ_12197 | -2.787464523 | 0.034116 | down | ENSG00000257923 | CUX1 |
| CIRI_circ_4415 | -2.790358592 | 0.031486 | down | ENSG00000115970 | THADA |
| CIRI_circ_7259 | -2.796843407 | 0.041634 | down | ENSG00000147649 | MTDH |
| CIRI_circ_9541 | -2.810372077 | 0.010713 | down | ENSG00000065833 | ME1 |
| CIRI_circ_12874 | -2.83308905 | 0.042484 | down | ENSG00000169902 | TPST1 |
| CIRI_circ_3905 | -2.841448427 | 0.042003 | down | ENSG00000073921 | PICALM |
| CIRI_circ_12684 | -2.84763569 | 0.044804 | down | ENSG00000135775 | COG2 |
| CIRI_circ_5087 | -2.851417982 | 0.021537 | down | ENSG00000057663 | ATG5 |
| CIRI_circ_9731 | -2.869700455 | 0.040064 | down | ENSG00000082701 | GSK3B |
| CIRI_circ_2235 | -2.872530653 | 0.042935 | down | ENSG00000177119 | ANO6 |
| CIRI_circ_15642 | -2.902922045 | 0.035523 | down | ENSG00000088179 | PTPN4 |
| CIRI_circ_924 | -2.908619881 | 0.007036 | down | ENSG00000161813 | LARP4 |
| CIRI_circ_15042 | -2.917887356 | 0.033719 | down | ENSG00000169398 | PTK2 |
| CIRI_circ_15198 | -2.918608508 | 0.036561 | down | ENSG00000138674 | SEC31A |
| CIRI_circ_13720 | -2.924026932 | 0.031961 | down | ENSG00000162415 | ZSWIM5 |
| CIRI_circ_16330 | -2.927688713 | 0.049425 | down | ENSG00000148516 | ZEB1 |
| CIRI_circ_1232 | -2.954160269 | 0.049834 | down | ENSG00000085511 | MAP3K4 |
| CIRI_circ_4863 | -2.965975772 | 6.03E-06 | down | ENSG00000184588 | PDE4B |
| CIRI_circ_13228 | -2.981719115 | 0.014826 | down | ENSG00000151466 | SCLT1 |
| CIRI_circ_3913 | -2.985085136 | 0.048696 | down | ENSG00000088179 | PTPN4 |
| CIRI_circ_681 | -3.010037676 | 0.016557 | down | ENSG00000136169 | SETDB2 |
| CIRI_circ_2798 | -3.037863366 | 0.025201 | down | ENSG00000175356 | SCUBE2 |
| CIRI_circ_11596 | -3.038009301 | 0.001362 | down | ENSG00000114796 | KLHL24 |
| CIRI_circ_3972 | -3.038731487 | 0.008222 | down | ENSG00000232855 | AF165147.1 |
| CIRI_circ_12721 | -3.065255983 | 0.022492 | down | ENSG00000168852 | TPTE2P5 |
| CIRI_circ_8106 | -3.066678917 | 0.040687 | down | ENSG00000270316 | BORCS7-ASMT |
| CIRI_circ_2072 | -3.074882696 | 0.017283 | down | ENSG00000064115 | TM7SF3 |
| CIRI_circ_4871 | -3.075773999 | 0.010573 | down | ENSG00000005700 | IBTK |
| CIRI_circ_13879 | -3.090775166 | 0.039547 | down | ENSG00000187240 | DYNC2H1 |
| CIRI_circ_596 | -3.141871405 | 0.011923 | down | ENSG00000031003 | FAM13B |
| CIRI_circ_2521 | -3.143638505 | 0.017562 | down | ENSG00000283563 | AC098650.1 |
| CIRI_circ_5501 | -3.147056419 | 0.026098 | down | ENSG00000197603 | CPLANE1 |
| CIRI_circ_10321 | -3.148511857 | 0.02442 | down | ENSG00000146072 | TNFRSF21 |
| CIRI_circ_10049 | -3.201033261 | 0.009521 | down | ENSG00000143842 | SOX13 |
| CIRI_circ_1568 | -3.216755146 | 0.025657 | down | ENSG00000163935 | SFMBT1 |
| CIRI_circ_13991 | -3.247002521 | 0.016057 | down | ENSG00000133106 | EPSTI1 |
| CIRI_circ_12810 | -3.252650375 | 0.015417 | down | ENSG00000284741 | PDE11A |
| CIRI_circ_1529 | -3.281195759 | 0.007327 | down | ENSG00000285943 | AC112128.1 |
| CIRI_circ_5640 | -3.281804296 | 0.042801 | down | ENSG00000197702 | PARVA |
| CIRI_circ_4830 | -3.320304921 | 0.033457 | down | ENSG00000135333 | EPHA7 |
| CIRI_circ_1469 | -3.366732107 | 0.047455 | down | ENSG00000080815 | PSEN1 |
| CIRI_circ_5475 | -3.367860763 | 0.046517 | down | ENSG00000078747 | ITCH |
| CIRI_circ_454 | -3.406669693 | 0.041446 | down | ENSG00000012983 | MAP4K5 |
| CIRI_circ_16143 | -3.41433076 | 0.041419 | down | ENSG00000159322 | ADPGK |
| CIRI_circ_4472 | -3.423767552 | 0.044103 | down | ENSG00000179912 | R3HDM2 |
| CIRI_circ_11305 | -3.427781438 | 0.007193 | down | ENSG00000135775 | COG2 |
| CIRI_circ_3507 | -3.431504647 | 0.040524 | down | ENSG00000118961 | LDAH |
| CIRI_circ_4930 | -3.439164504 | 0.00216 | down | ENSG00000154655 | L3MBTL4 |
| CIRI_circ_3370 | -3.445040043 | 0.037593 | down | ENSG00000140694 | PARN |
| CIRI_circ_2177 | -3.45130383 | 0.002388 | down | ENSG00000019995 | ZRANB1 |
| CIRI_circ_10170 | -3.473089816 | 0.034688 | down | ENSG00000111058 | ACSS3 |
| CIRI_circ_2040 | -3.48340927 | 0.038098 | down | ENSG00000101596 | SMCHD1 |
| CIRI_circ_4860 | -3.494958786 | 0.036843 | down | ENSG00000171953 | ATPAF2 |
| CIRI_circ_12313 | -3.496465072 | 0.034418 | down | ENSG00000101596 | SMCHD1 |
| CIRI_circ_13479 | -3.506092059 | 0.00533 | down | ENSG00000084676 | NCOA1 |
| CIRI_circ_5674 | -3.507399803 | 0.032941 | down | ENSG00000169398 | PTK2 |
| CIRI_circ_3306 | -3.511219205 | 0.005585 | down | ENSG00000066468 | FGFR2 |
| CIRI_circ_11011 | -3.513566648 | 0.04985 | down | ENSG00000072736 | NFATC3 |
| CIRI_circ_13096 | -3.515510076 | 0.000508 | down | ENSG00000232855 | AF165147.1 |
| CIRI_circ_2626 | -3.520626087 | 0.048294 | down | ENSG00000117020 | AKT3 |
| CIRI_circ_15770 | -3.521238339 | 0.033954 | down | ENSG00000092148 | HECTD1 |
| CIRI_circ_2612 | -3.538719805 | 0.016782 | down | ENSG00000150760 | DOCK1 |
| CIRI_circ_12923 | -3.542798478 | 0.048505 | down | ENSG00000156502 | SUPV3L1 |
| CIRI_circ_6847 | -3.542854617 | 0.029962 | down | ENSG00000122545 | SEPTIN7 |
| CIRI_circ_7530 | -3.551416375 | 0.048258 | down | ENSG00000146463 | ZMYM4 |
| CIRI_circ_4123 | -3.553053677 | 0.031885 | down | ENSG00000135040 | NAA35 |
| CIRI_circ_9000 | -3.553811436 | 0.048459 | down | ENSG00000144357 | UBR3 |
| CIRI_circ_6248 | -3.55441689 | 0.02905 | down | ENSG00000115419 | GLS |
| CIRI_circ_8538 | -3.570663911 | 0.045574 | down | ENSG00000152894 | PTPRK |
| CIRI_circ_14164 | -3.573318916 | 0.047972 | down | ENSG00000008311 | AASS |
| CIRI_circ_15999 | -3.583626921 | 0.014688 | down | ENSG00000114796 | KLHL24 |
| CIRI_circ_15212 | -3.584493384 | 0.044126 | down | ENSG00000197548 | ATG7 |
| CIRI_circ_15032 | -3.586544929 | 0.044491 | down | ENSG00000052126 | PLEKHA5 |
| CIRI_circ_1391 | -3.601243119 | 0.030838 | down | ENSG00000166377 | ATP9B |
| CIRI_circ_11191 | -3.613057544 | 0.040649 | down | ENSG00000075420 | FNDC3B |
| CIRI_circ_2342 | -3.613561116 | 0.040512 | down | ENSG00000131375 | CAPN7 |
| CIRI_circ_8509 | -3.616960323 | 0.024588 | down | ENSG00000099956 | SMARCB1 |
| CIRI_circ_7965 | -3.618866443 | 0.043724 | down | ENSG00000157540 | DYRK1A |
| CIRI_circ_12334 | -3.621057773 | 0.041793 | down | ENSG00000078124 | ACER3 |
| CIRI_circ_4107 | -3.643897722 | 0.0431 | down | ENSG00000011114 | BTBD7 |
| CIRI_circ_11106 | -3.655352362 | 0.038836 | down | ENSG00000066468 | FGFR2 |
| CIRI_circ_14968 | -3.655583886 | 0.041906 | down | ENSG00000135597 | REPS1 |
| CIRI_circ_11769 | -3.660309275 | 0.004804 | down | ENSG00000133424 | LARGE1 |
| CIRI_circ_7677 | -3.674078019 | 0.00895 | down | ENSG00000133392 | MYH11 |
| CIRI_circ_4520 | -3.675959867 | 9.35E-05 | down | ENSG00000100473 | COCH |
| CIRI_circ_15312 | -3.67607313 | 0.020754 | down | ENSG00000124535 | WRNIP1 |
| CIRI_circ_155 | -3.683393981 | 0.019723 | down | ENSG00000164253 | WDR41 |
| CIRI_circ_10384 | -3.68915119 | 0.038915 | down | ENSG00000115841 | RMDN2 |
| CIRI_circ_8246 | -3.689290926 | 0.034146 | down | ENSG00000071205 | ARHGAP10 |
| CIRI_circ_14263 | -3.69614817 | 0.036138 | down | ENSG00000237975 | FLG-AS1 |
| CIRI_circ_9816 | -3.710609719 | 0.019098 | down | ENSG00000104218 | CSPP1 |
| CIRI_circ_6033 | -3.713280754 | 0.022574 | down | ENSG00000125354 | SEPTIN6 |
| CIRI_circ_3675 | -3.715573382 | 0.037919 | down | ENSG00000236532 | LINC01695 |
| CIRI_circ_2096 | -3.721382059 | 0.03541 | down | ENSG00000171466 | ZNF562 |
| CIRI_circ_13015 | -3.724128006 | 0.021998 | down | ENSG00000152527 | PLEKHH2 |
| CIRI_circ_5094 | -3.7288856 | 0.017604 | down | ENSG00000169375 | SIN3A |
| CIRI_circ_15371 | -3.753463185 | 0.01776 | down | ENSG00000241962 | AC079447.1 |
| CIRI_circ_3659 | -3.754173857 | 0.034873 | down | ENSG00000169398 | PTK2 |
| CIRI_circ_10064 | -3.774736529 | 0.015338 | down | ENSG00000086200 | IPO11 |
| CIRI_circ_5025 | -3.777660172 | 0.033594 | down | ENSG00000109381 | ELF2 |
| CIRI_circ_13838 | -3.781138412 | 0.002033 | down | ENSG00000154655 | L3MBTL4 |
| CIRI_circ_14498 | -3.792417908 | 0.028594 | down | ENSG00000165338 | HECTD2 |
| CIRI_circ_3614 | -3.801372185 | 0.01597 | down | ENSG00000122507 | BBS9 |
| CIRI_circ_8845 | -3.802649743 | 0.027387 | down | ENSG00000172915 | NBEA |
| CIRI_circ_5052 | -3.827361671 | 0.013389 | down | ENSG00000140153 | WDR20 |
| CIRI_circ_11188 | -3.837110922 | 0.008238 | down | ENSG00000242441 | GTF2A1L |
| CIRI_circ_2329 | -3.84922584 | 0.027329 | down | ENSG00000175029 | CTBP2 |
| CIRI_circ_8851 | -3.854838125 | 0.011908 | down | ENSG00000118454 | ANKRD13C |
| CIRI_circ_1223 | -3.856516678 | 0.026119 | down | ENSG00000106443 | PHF14 |
| CIRI_circ_2559 | -3.85794528 | 0.022323 | down | ENSG00000067141 | NEO1 |
| CIRI_circ_5559 | -3.860332927 | 0.00148 | down | ENSG00000123106 | CCDC91 |
| CIRI_circ_11213 | -3.866846004 | 0.024052 | down | ENSG00000141627 | DYM |
| CIRI_circ_5757 | -3.881400494 | 0.02076 | down | ENSG00000197121 | PGAP1 |
| CIRI_circ_7206 | -3.882852502 | 0.0117 | down | ENSG00000105855 | ITGB8 |
| CIRI_circ_4153 | -3.891412866 | 0.020177 | down | ENSG00000049618 | ARID1B |
| CIRI_circ_6371 | -3.892267885 | 0.001055 | down | ENSG00000025796 | SEC63 |
| CIRI_circ_11674 | -3.899737067 | 0.010233 | down | ENSG00000142453 | CARM1 |
| CIRI_circ_10500 | -3.900554027 | 0.011191 | down | ENSG00000183346 | CABCOCO1 |
| CIRI_circ_1048 | -3.901600649 | 0.010121 | down | ENSG00000165521 | EML5 |
| CIRI_circ_11355 | -3.908254432 | 0.021253 | down | ENSG00000100934 | SEC23A |
| CIRI_circ_8158 | -3.915677822 | 0.019348 | down | _ | _ |
| CIRI_circ_3029 | -3.916658671 | 0.027934 | down | ENSG00000231887 | PRH1 |
| CIRI_circ_3405 | -3.91702681 | 0.019414 | down | ENSG00000197121 | PGAP1 |
| CIRI_circ_1385 | -3.919500809 | 0.009142 | down | ENSG00000145293 | ENOPH1 |
| CIRI_circ_3000 | -3.923103123 | 0.009472 | down | ENSG00000137710 | RDX |
| CIRI_circ_1733 | -3.925159495 | 0.009345 | down | ENSG00000172795 | DCP2 |
| CIRI_circ_1244 | -3.943630402 | 0.018707 | down | ENSG00000101639 | CEP192 |
| CIRI_circ_114 | -3.945039228 | 0.033603 | down | ENSG00000204644 | ZFP57 |
| CIRI_circ_1961 | -3.947313076 | 0.010085 | down | ENSG00000111880 | RNGTT |
| CIRI_circ_13062 | -3.95315503 | 0.032704 | down | ENSG00000114796 | KLHL24 |
| CIRI_circ_11655 | -3.955778723 | 0.010631 | down | ENSG00000124789 | NUP153 |
| CIRI_circ_6793 | -3.956263732 | 0.034122 | down | _ | _ |
| CIRI_circ_9137 | -3.956982363 | 0.00913 | down | ENSG00000108055 | SMC3 |
| CIRI_circ_14673 | -3.9636304 | 0.033764 | down | ENSG00000197776 | KLHDC1 |
| CIRI_circ_15058 | -3.964269841 | 0.01776 | down | ENSG00000144857 | BOC |
| CIRI_circ_9790 | -3.983378671 | 0.00887 | down | ENSG00000064933 | PMS1 |
| CIRI_circ_14248 | -3.984804451 | 0.016601 | down | ENSG00000153993 | SEMA3D |
| CIRI_circ_11207 | -3.991865345 | 0.01566 | down | ENSG00000118420 | UBE3D |
| CIRI_circ_26 | -3.992797964 | 0.031471 | down | ENSG00000087206 | UIMC1 |
| CIRI_circ_9312 | -3.995755061 | 0.017126 | down | ENSG00000138398 | PPIG |
| CIRI_circ_11646 | -3.998453855 | 0.016498 | down | ENSG00000104375 | STK3 |
| CIRI_circ_14067 | -4.006472871 | 0.017354 | down | ENSG00000068878 | PSME4 |
| CIRI_circ_11648 | -4.008402102 | 0.018056 | down | ENSG00000225689 | AL008633.1 |
| CIRI_circ_9841 | -4.011535166 | 0.007157 | down | ENSG00000110422 | HIPK3 |
| CIRI_circ_3482 | -4.012617775 | 0.00773 | down | ENSG00000257103 | LSM14A |
| CIRI_circ_2972 | -4.025677664 | 0.017942 | down | ENSG00000158966 | CACHD1 |
| CIRI_circ_1986 | -4.03139897 | 0.006529 | down | ENSG00000061987 | MON2 |
| CIRI_circ_3759 | -4.038303857 | 0.018046 | down | ENSG00000010803 | SCMH1 |
| CIRI_circ_11706 | -4.062938926 | 0.015452 | down | ENSG00000115556 | PLCD4 |
| CIRI_circ_12135 | -4.066840503 | 0.012315 | down | ENSG00000205339 | IPO7 |
| CIRI_circ_8769 | -4.077097678 | 0.0172 | down | ENSG00000116962 | NID1 |
| CIRI_circ_9118 | -4.082126186 | 0.013735 | down | ENSG00000198947 | DMD |
| CIRI_circ_3930 | -4.102675365 | 0.011397 | down | ENSG00000103404 | USP31 |
| CIRI_circ_2433 | -4.113327565 | 0.005319 | down | ENSG00000148634 | HERC4 |
| CIRI_circ_13022 | -4.149282707 | 0.010714 | down | ENSG00000163655 | GMPS |
| CIRI_circ_2273 | -4.152884406 | 0.010809 | down | ENSG00000171100 | MTM1 |
| CIRI_circ_9383 | -4.186090755 | 0.020164 | down | ENSG00000185760 | KCNQ5 |
| CIRI_circ_1006 | -4.190367838 | 0.018967 | down | ENSG00000168487 | BMP1 |
| CIRI_circ_12078 | -4.19123386 | 0.004074 | down | ENSG00000111728 | ST8SIA1 |
| CIRI_circ_9863 | -4.211298746 | 0.00472 | down | ENSG00000213079 | SCAF8 |
| CIRI_circ_5338 | -4.223857232 | 0.00369 | down | ENSG00000167306 | MYO5B |
| CIRI_circ_1619 | -4.224305832 | 0.008344 | down | ENSG00000071205 | ARHGAP10 |
| CIRI_circ_2939 | -4.230836176 | 0.008191 | down | ENSG00000127720 | METTL25 |
| CIRI_circ_2257 | -4.235125122 | 0.008412 | down | ENSG00000109339 | MAPK10 |
| CIRI_circ_11338 | -4.297218133 | 0.003631 | down | ENSG00000114439 | BBX |
| CIRI_circ_5123 | -4.302327538 | 0.006892 | down | ENSG00000122507 | BBS9 |
| CIRI_circ_1574 | -4.316482225 | 0.01457 | down | ENSG00000145012 | LPP |
| CIRI_circ_5925 | -4.321077701 | 0.005882 | down | ENSG00000166979 | EVA1C |
| CIRI_circ_5709 | -4.342391414 | 0.002926 | down | ENSG00000165487 | MICU2 |
| CIRI_circ_10031 | -4.360871466 | 0.003188 | down | ENSG00000183666 | GUSBP1 |
| CIRI_circ_10791 | -4.365178286 | 0.002259 | down | ENSG00000152683 | SLC30A6 |
| CIRI_circ_7818 | -4.371033427 | 0.00502 | down | ENSG00000153827 | TRIP12 |
| CIRI_circ_3907 | -4.373489144 | 0.002101 | down | ENSG00000123684 | LPGAT1 |
| CIRI_circ_8054 | -4.377456391 | 0.001853 | down | ENSG00000185842 | DNAH14 |
| CIRI_circ_14439 | -4.397100475 | 0.001728 | down | ENSG00000111666 | CHPT1 |
| CIRI_circ_1712 | -4.411822495 | 0.005008 | down | ENSG00000154310 | TNIK |
| CIRI_circ_11602 | -4.417276131 | 0.001569 | down | ENSG00000074657 | ZNF532 |
| CIRI_circ_3656 | -4.4559119 | 0.004268 | down | ENSG00000151572 | ANO4 |
| CIRI_circ_12548 | -4.500859132 | 0.005056 | down | ENSG00000146151 | HMGCLL1 |
| CIRI_circ_9821 | -4.509137378 | 0.003194 | down | ENSG00000179397 | CATSPERE |
| CIRI_circ_14484 | -4.52348647 | 0.003541 | down | ENSG00000173175 | ADCY5 |
| CIRI_circ_6528 | -4.560349712 | 0.001067 | down | ENSG00000265354 | TIMM23 |
| CIRI_circ_15931 | -4.569269771 | 0.00262 | down | ENSG00000140563 | MCTP2 |
| CIRI_circ_12106 | -4.590904121 | 0.00245 | down | ENSG00000124440 | HIF3A |
| CIRI_circ_10552 | -4.597425979 | 0.002579 | down | ENSG00000071205 | ARHGAP10 |
| CIRI_circ_15859 | -4.641027706 | 0.002574 | down | ENSG00000288637 | AC013477.2 |
| CIRI_circ_15060 | -4.646189051 | 0.001203 | down | ENSG00000180488 | MIGA1 |
| CIRI_circ_224 | -4.65437092 | 0.002155 | down | ENSG00000133392 | MYH11 |
| CIRI_circ_12305 | -4.772913682 | 0.001365 | down | ENSG00000071205 | ARHGAP10 |
| CIRI_circ_9642 | -4.820706899 | 0.000208 | down | ENSG00000242086 | MUC20-OT1 |
| CIRI_circ_9880 | -4.851191389 | 0.000193 | down | ENSG00000119906 | SLF2 |
| CIRI_circ_5268 | -4.851674214 | 0.001003 | down | ENSG00000175662 | TOM1L2 |
| CIRI_circ_6764 | -4.855734548 | 0.001012 | down | ENSG00000119787 | ATL2 |
| CIRI_circ_9213 | -5.019116673 | 0.000191 | down | ENSG00000153993 | SEMA3D |
| CIRI_circ_1785 | -5.089190109 | 0.000555 | down | ENSG00000107518 | ATRNL1 |
| CIRI_circ_9133 | -5.257128916 | 6.49E-05 | down | ENSG00000138119 | MYOF |
| CIRI_circ_10071 | -5.593717786 | 0.000133 | down | _ | _ |
| CIRI_circ_2008 | -5.819757158 | 1.07E-05 | down | ENSG00000107518 | ATRNL1 |
| CIRI_circ_1701 | -7.501155938 | 0.013208 | down | ENSG00000107077 | KDM4C |

**Table S8. Association between circSPON2 and clinicopathological features of PCa patients.**

| Characteristics | Cases (%) | CircSPON2 expression | *P* value |
| --- | --- | --- | --- |
| Age |  |  |  |
| <70 | 110 (44.35%) | 8.76 ± 4.95 | 0.7134 |
| ≥70 | 138 (55.65%) | 8.99 ± 4.84 |  |
| Gleason score |  |  |  |
| 5-7 | 148 (59.68%) | 6.28 ± 3.37 | **0.000*** |
| 8-10 | 100 (40.32%) | 12.77 ± 4.15 |  |
| T stage |  |  |  |
| T1-2 | 145 (58.47%) | 6.53 ± 3.51 | **0.000*** |
| T3-4 | 103 (41.32%) | 12.22 ± 4.60 |  |
| Lymph node metastasis |  |  |  |
| N0 | 87 (35.08%) | 8.45 ± 4.84 | 0.2928 |
| N+ | 161 (64.92%) | 9.13 ± 4.90 |  |
| Distant metastasis |  |  |  |
| M0 | 228 (91.94%) | 8.77 ± 4.87 | 0.3024 |
| M1 | 18 (7.26%) | 9.99 ± 4.33 |  |

**P* < 0.05 is considered significant.
